# Ag(I)/K_2_S_2_O_8_‑Mediated Selective Oxidation of Ynamide-Yne via Structural Reshuffling and Consecutive *N*‑Desulfonylation

**DOI:** 10.1021/acs.joc.5c01007

**Published:** 2025-07-08

**Authors:** Mohana Reddy Mutra, T. L. Chandana, Tzu-Pin Wang, Jeh-Jeng Wang

**Affiliations:** † Department of Medicinal and Applied Chemistry, 38023 Kaohsiung Medical University, No. 100, Shiquan first Rd, San-min District, Kaohsiung City 807, Taiwan; ‡ Department of Medical Research, Kaohsiung Medical University Hospital, No. 100, Tzyou first Rd, Sanmin District, Kaohsiung City 807, Taiwan

## Abstract

Herein, we developed an intramolecular Ag­(I)/K_2_S_2_O_8_-mediated selective oxidation of alkyne in the ynamide-yne framework. Through structural reshuffling and consecutive *N*-desulfonylation, this process produces multisubstituted indoles (1,2-dicarbonyl). The developed intramolecular methodology exhibits remarkable advantages, including regioselective and chemoselective alkyne oxidation, the use of K_2_S_2_O_8_ as an oxidant and oxygen source, multiple bond cleavages (N–C­(sp), O–SO_3_^–^, N–SO_2_R), and the formation of multiple new bonds (two C=O, two C­(sp^2^)–C­(sp^2^), and one N–C­(sp^2^)) in a one-pot reaction. Moreover, this protocol is applicable to gram-scale synthesis and enables further synthetic transformations of the products.

## Graphical abstract

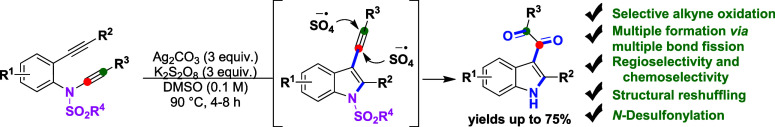

## Introduction

Ynamides are highly unsymmetrical, electron-rich nitrogen-containing alkynes that have found extensive use in the construction of diverse nitrogen-containing compounds.^1^ The polarization of the α and β carbons in ynamides, influenced by the electron-withdrawing amide group, facilitates the development of highly efficient regioselective and chemoselective transformations.^1^ Well-established strategies for the oxidation of ynamides typically yield α-ketoimide compounds.^2^ In recent literature, several protocols have been developed for ynamide oxidation using both metal and metal-free methodologies.^2^ Most of these transformations generate α-oxo carbene or keteniminium intermediates in situ, aided by transition-metal catalysts or Brønsted acids, which are subsequently attacked by external counterions to produce the corresponding α-ketoimides. In this context, Hwang reported a photochemical aerobic oxidation of simple ynamides using copper­(I) chloride as a catalyst (Scheme 1A­(1)).^2a^ Ye achieved the synthesis of α-ketoimides from simple ynamides using *N*-oxides as oxidants under copper catalysis (Scheme 1A­(2)).^2b^ Xu developed a microwave-assisted oxidation of ynamides into *N*-sulfonyloxoacetamides using dimethyl sulfoxide (DMSO) at extremely high temperatures (Scheme 1A­(3)).^2c^ More recently, Rong described a mercury-catalyzed synthesis of α-ketoimides from ynamides using 2-chloropyridine *N*-oxide (Scheme 1A­(4)).^2d^ Despite their respective advantages, all of these methods have certain drawbacks, including the exclusive use of simple ynamides, lack of regioselectivity and chemoselectivity, ionic pathway mechanisms, reliance on well-known oxidation conditions, and the need for harsh reaction environments.

**Scheme 1:**
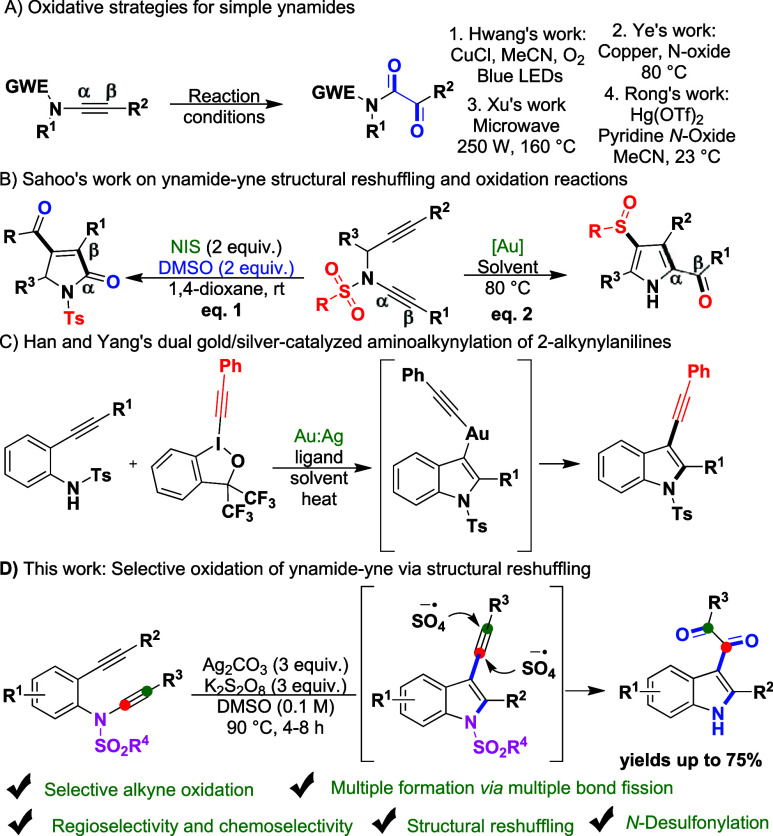
(A) Oxidative Strategies for Simple Ynamides; (B) Sahoo’s Work on Structural Reshuffling and Oxidation of Ynamide-Ynes; (C) Dual-Catalyzed (Au and Ag) Construction of Multi-Substituted Indoles from 2-Alkynylanilines; (D) Selective Alkyne Oxidation in Ynamide-Ynes via Structural Reshuffling and Consecutive *N*-Desulfonylation to Yield Multi-Substituted Indoles

Considering the reactivity of simple ynamides, ynamide-ynes present additional challenges, including issues of regioselectivity, chemoselectivity, and difficulty in obtaining a single product due to their multiple reactive sites.^3^ Sahoo synthesized pyrrolidones and spiro-pyrrolidones from nitrogen-tethered ynamide-ynes through oxidation using *N*-iodosuccinimide (NIS) and DMSO (Scheme 2B, eq 1).^3a^ The same group also developed an oxidative, regioselective sulfonyl/sulfinyl migration–cycloisomerization cascade reaction using the same ynamide-yne precursors in the presence of a gold (Au) catalyst (Scheme 2B, eq 2).^3b^ Very recently, Han and Yang synthesized multisubstituted indoles from 2-alkynylanilines using hypervalent iodine­(III) reagents under dual gold/silver catalysis in open-air conditions (Scheme 1C).^4^ Despite their excellent oxidative strategies, these methods have drawbacks, such as the absence of N–C­(sp) bond cleavage, the use of expensive metal catalysts, oxidation of only a single carbon center, and nonradical mechanisms. Our group has previously demonstrated the use of ynamide-ynes/ynamides in the synthesis of nitrogen-containing bioactive compounds.^5^ Herein, we report an intramolecular Ag­(I)/K_2_S_2_O_8_-mediated selective oxidation of the alkyne in ynamide-ynes via skeletal reorganization and simultaneous *N*-desulfonylation to construct indole-tethered 1,2-dicarbonyl compounds (Scheme 1D). Previously, a few intermolecular strategies have been developed for the synthesis of 3-acylindoles (1–2 examples), such as the use of *N*-phenylamidines with α-chloroketones in the presence of [Cp*RhCl_2_]_2_ and AgOAc,^6a^ or 2-alkynylanilines with α-amino carbonyl compounds under Pd­(OAc)_2_/Cu­(OAc)_2_ catalysis.^6b^ In contrast, our newly developed intramolecular methodology exhibits significant advantages, including high regioselectivity and chemoselectivity, selective oxidation of the ynamide-alkyne via a structural reshuffling process, multiple bond cleavages (N–C­(sp), two O–SO_3_^–^, and N–SO_2_R), and formation of multiple new bonds (two C=O, two C­(sp^2^)–C­(sp^2^), and one N–C­(sp^2^)) in a single-pot reaction. Moreover, this protocol is applicable to gram-scale synthesis and facilitates further synthetic elaboration of the products.

## Results and Discussion

We initiated our optimization studies using 4-methyl-*N*-(phenylethynyl)-*N*-(2-(phenylethynyl)­phenyl)­benzene­sulfon­amide (**1a**) under AgNO_3_/K_2_S_2_O_8_-mediated^7^ conditions in 1,2-dichloroethane (DCE) at 90 °C. This led to a selective oxidative transformation of the ynamide–alkyne system via structural reshuffling, affording 1-phenyl-2-(2-phenyl-1-tosylindol-3-yl)­ethane-1,2-dione (**2a**) as the major product in 45% yield, along with 1-phenyl-2-(2-phenyl-1*H*-indol-3-yl)­ethane-1,2-dione (**2**) as a minor desulfonylated product in 5% yield (Table 1, entry 1). To improve the efficiency, various solvents were evaluated, including water, 1,4-dioxane, PEG-400, ethanol, toluene, anisole, and dimethyl sulfoxide (DMSO) (Table 1, entries 2–8). Among these, DMSO significantly enhanced the yield of **2a** to 58%. Replacement of K_2_S_2_O_8_ with other oxidants led to diminished yields (Table 1, entries 9 and 10), highlighting its crucial role in this transformation. Next, we assessed alternative silver salts (Ag_2_CO_3_, AgOCOCF_3_, AgOTf) and other Lewis acids (Cu­(OAc)_2_, FeCl_3_) (Table 1, entries 11–15). Notably, Ag_2_CO_3_ provided the desired product **2** in 60% yield. Further increasing the loading of both Ag_2_CO_3_ and K_2_S_2_O_8_ improved the yield of **2** to 73% (Table 1, entry 16). Omission of either Ag_2_CO_3_ or K_2_S_2_O_8_ resulted in complete suppression or only trace amounts of product formation, underscoring their synergistic necessity (Table 1, entries 17 and 18).

**Table 1:**
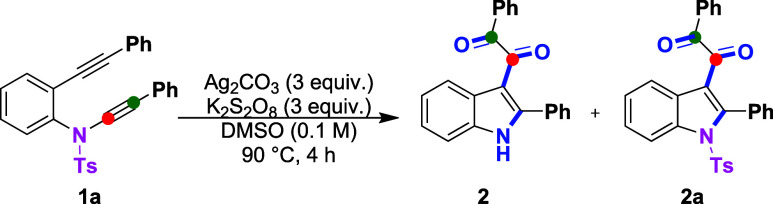
Screening of the Reaction Conditions^a^

entry	metal source (*x* equiv)	oxidant (*y* equiv)	solvent (time in hours)	yield^b^ (**2**/**2a**)
1	AgNO_3_ (2)	K_2_S_2_O_8_ (2)	DCE (15 h)	5/45
2	AgNO_3_ (2)	K_2_S_2_O_8_ (2)	1,4-dioxane (15 h)	trace/25
3	AgNO_3_ (2)	K_2_S_2_O_8_ (2)	PEG-400 (15 h)	trace/<5
4	AgNO_3_ (2)	K_2_S_2_O_8_ (2)	EtOH (15 h)	trace/<5
5	AgNO_3_ (2)	K_2_S_2_O_8_ (2)	H_2_O (15 h)	0/0
6	AgNO_3_ (2)	K_2_S_2_O_8_ (2)	toluene (15 h)	<5/23
7	AgNO_3_ (2)	K_2_S_2_O_8_ (2)	anisole (15 h)	0/<5
8	AgNO_3_ (2)	K_2_S_2_O_8_ (2)	DMSO (5 h)	8/58
9	AgNO_3_ (2)	oxone (2)	DMSO (5 h)	0/20
10	AgNO_3_ (2)	70% aq. TBHP (2)	DMSO (5 h)	<5/40
11	Ag_2_CO_3_ (2)	K_2_S_2_O_8_ (2)	DMSO (5 h)	60/6
12	AgOCOCF_3_ (2)	K_2_S_2_O_8_ (2)	DMSO (5 h)	25/8
13	AgOTf (2)	K_2_S_2_O_8_ (2)	DMSO (5 h)	20/27
14	Cu(OAc)_2_ (2)	K_2_S_2_O_8_ (2)	DMSO (5 h)	-/15
15	FeCl_3_ (2)	K_2_S_2_O_8_ (2)	DMSO (5 h)	trace/50
16	Ag_2_CO_3_ (3)	K_2_S_2_O_8_ (3)	DMSO (4 h)	73/6
17		K_2_S_2_O_8_ (3)	DMSO (4 h)	trace/trace
18	Ag_2_CO_3_ (3)		DMSO (4 h)	0/0
19^c^	Ag_2_CO_3_ (3)	K_2_S_2_O_8_ (3)	DMSO (4 h)	61/<5
20^d^	Ag_2_CO_3_ (3)	K_2_S_2_O_8_ (3)	DMSO (4 h)	0/0
21	Ag_2_CO_3_ (4)	K_2_S_2_O_8_ (4)	DMSO (4 h)	75/7
22	Ag_2_CO_3_ (5)	K_2_S_2_O_8_ (5)	DMSO (4 h)	72/5

a Reaction condition: **1a** (0.15 mmol), Ag_2_CO_3_ (0.45 mmol), K_2_S_2_O_8_ (0.45 mmol), DMSO (0.1 M) and stirred for 4 h at 90 °C.

b Isolated yields.

c Reaction mixture heated at 130 °C.

d Reaction mixture stirred at 25 °C.

Temperature played a critical role: elevating the temperature to 130 °C decreased the yield (Table 1, entry 19), while conducting the reaction at room temperature (25 °C) led to no detectable product formation (Table 1, entry 20). Additionally, increasing the amounts of metal catalyst and oxidant to 4 and 5 equiv, respectively, gave similar product yields without significant improvement (Table 1, entries 20 and 21). Therefore, we selected 3 equiv of each as the optimal conditions for the reaction.

To evaluate the generality of this oxidative structural reshuffling reaction, we applied the optimized protocol to a diverse set of substituted ynamide-ynes (Table 2)­. Substrates bearing aryl group R^1^ with electron-donating or electron-withdrawing substituents—including hydrogen (**1a**), methyl (**1b**), ethyl (**1c**), and chlorine (**1d**)—underwent efficient transformation to furnish the corresponding diketone products **2**–**5** in 65%–73% yields. We next examined the electronic influence of substitutions at the R^2^ position. Ynamide-ynes bearing electron-rich aromatic groups such as *ortho*-methylphenyl (**1e**), *para*-methylphenyl (**1f**), *para*-ethylphenyl (**1g**), and *para*-methoxyphenyl (**1h**) provided products **6**–**9** in 61%–75% yields. Electron-deficient substrates, such as *meta*-chloro-phenyl (**1i**) and *para*-carboxymethylphenyl (**1j**), were also well tolerated, delivering products **10** and **11** in 56% and 66% yields, respectively. Substituent effects at the R^3^ position of the ynamide-yne were then explored. Substrates bearing *para*-methoxyphenyl (**1k**), *meta*-chlorophenyl (**1l**), *meta*-nitrophenyl (**1m**), and *para*-carboxymethylphenyl (**1n**) groups were successfully converted into products **12**–**15** in moderate yields (41%–62%). In contrast, attempts to synthesize products **16** and **17** from aliphatic R^3^-substituted ynamide-ynes (**1o** and **1p**) were unsuccessful, resulting in complex mixtures or unidentified byproducts. Additionally, efforts to prepare other aliphatic R^3^-substituted starting materials failed. In these cases, the formation of indole byproducts was observed, likely due to the lower reactivity of the corresponding alkynyl bromides. While phenyl-derived groups were the primary focus of this study, preliminary experiments with selected R^3^ heteroaryl substituents—such as 4-methyl-*N*-(2-(phenylethynyl)­phenyl)-*N*-(thiophen-2-ylethynyl)­benzenesulfonamide (**1q**)—resulted only in recovery of the starting material, with no observable product formation under the current conditions. We further extended the scope by modifying the R^4^ group on the sulfonamide moiety to evaluate the compatibility of the *N*-desulfonylation strategy. Aryl sulfonamides such as phenyl (**1aa**), *para*-methoxyphenyl (**1ab**), and *para*-*tert*-butylphenyl (**1ac**) afforded the desulfonylated product **2** in 59%–65% yields. Electron-withdrawing halogenated sulfonamides—including *para*-fluorophenyl (**1ad**), *para*-chlorophenyl (**1ae**), *para*-bromophenyl (**1af**), *para*-iodophenyl (**1ag**), and *para*-trifluoromethylphenyl (**1ah**)—also furnished product **2** in 50%–67% yields. Notably, the use of a sterically bulky naphthyl-substituted sulfonamide (**1ai**) yielded product **2** in 55% yield. Interestingly, the desulfonylation strategy was also effective with aliphatic sulfonyl groups such as methyl (**1aj**), ethyl (**1ak**), and cyclopropane (**1al**), affording product **2** in 51%–58% yields. Importantly, the sulfonamide moiety –SO_2_N­(CH_3_)_2_ (**1am**) also furnished product **2** in 48% yield. Based on all these observations, we conclude that the chemical and electronic variation in sulfonamide substituents does not restrict the reaction outcome. In all reactions, the mass balance is primarily attributed to incomplete conversion and the formation of side products, including *N*-tosyl-protected minor products and other minor species that are not readily isolable or fully characterized under the current conditions. Additionally, some substrates may undergo competing reaction pathways leading to these minor byproducts.

**Table 2:**
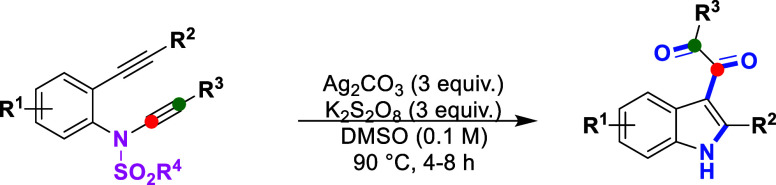

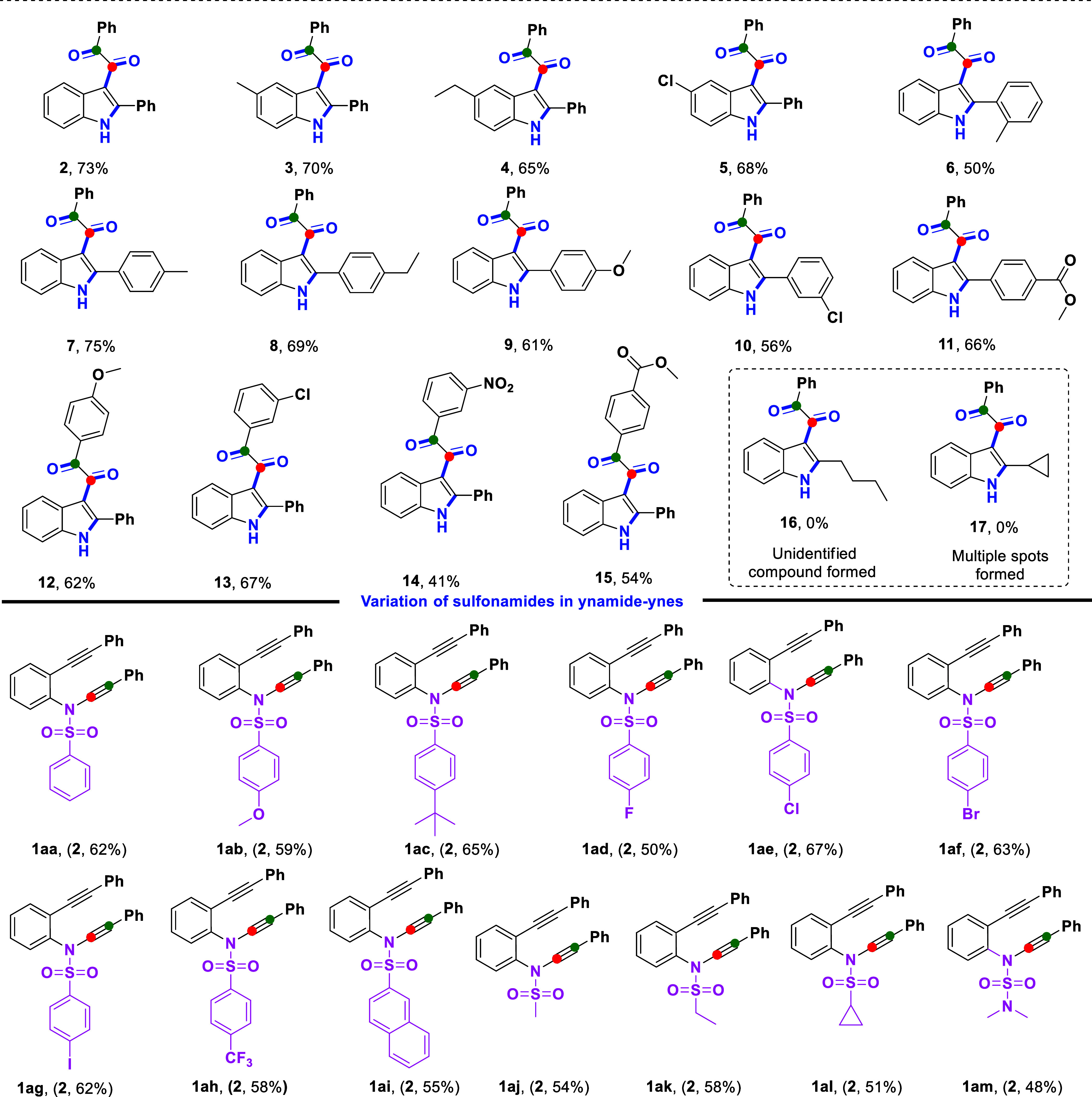
Scope of Ynamide-Ynes^a^

a Reaction conditions: **1** (0.15 mmol), Ag_2_CO_3_ (0.45 mmol), K_2_S_2_O_8_ (0.45 mmol), and DMSO (0.1 M) were heated at 90 °C for 4–8 h; isolated yields.

To underscore the significance of this transformation, we performed a gram-scale reaction followed by further synthetic elaboration of the product, as shown in Scheme 2A. Specifically, 1.0 g of substrate **1a** was subjected to the optimized reaction conditions, affording the desired product **2** in a satisfactory yield (66%). Subsequent treatment of **2** with sodium borohydride in ethanol furnished the reduced product **18** in 90% yield. Furthermore, reaction of **2** with *p*-toluenesulfonyl chloride in 60% NaH (oil) afforded the tosylated derivative, 1-phenyl-2-(2-phenyl-1-tosyl-1*H*-indol-3-yl)­ethane-1,2-dione (**2a**), in 65% yield.

**Scheme 2:**
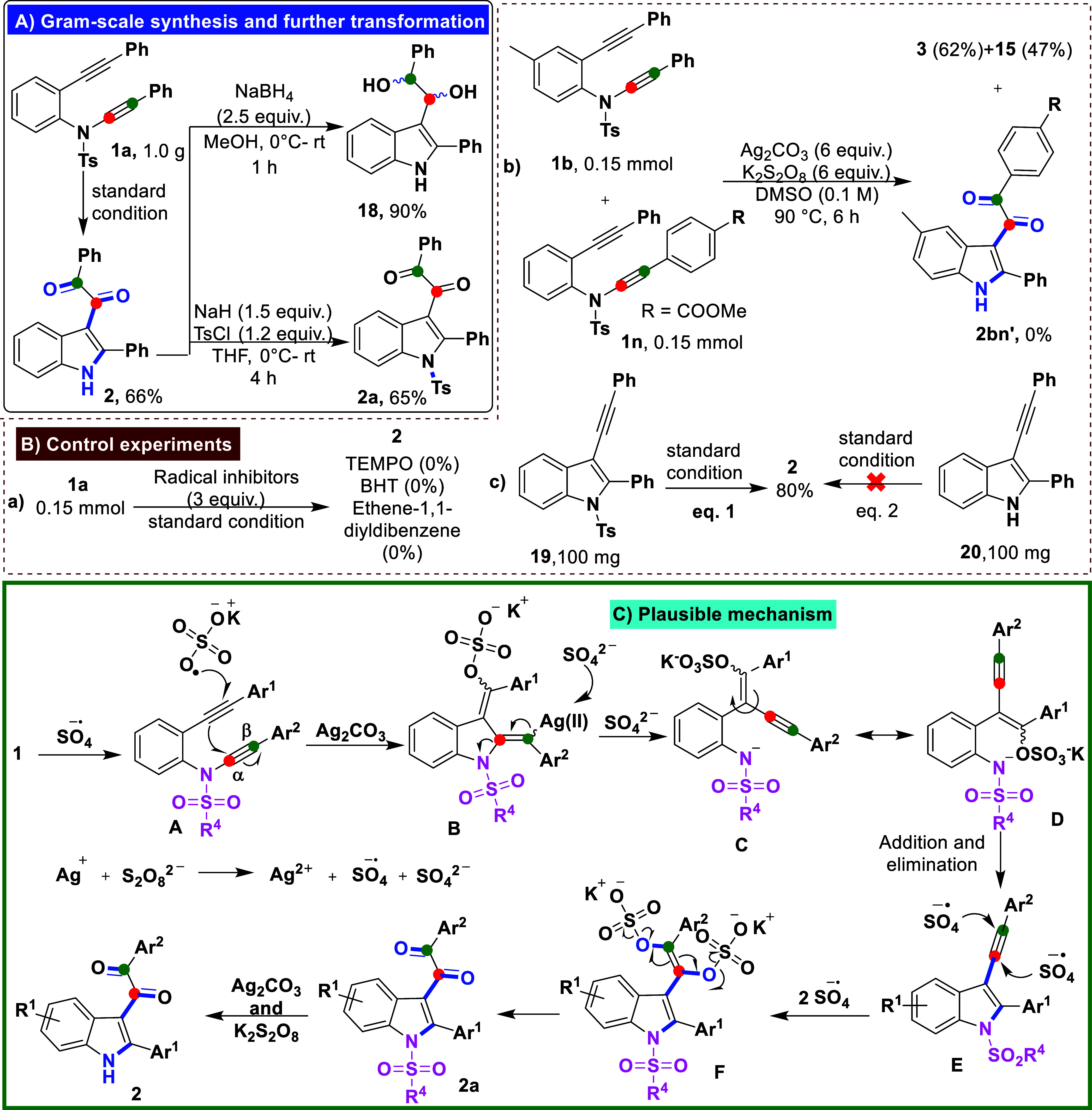
(A) Gram-Scale Synthesis and Subsequent Derivatization; (B) Control Experiments to Probe the Reaction Mechanism; (C) Proposed Mechanism Involving a Radical Pathway

To gain mechanistic insight into the transformation, a series of control experiments was conducted, as depicted in Scheme 2B. Reactions were carried out in the presence of various radical scavengers, including (2,2,6,6-tetramethylpiperidin-1-yl)­oxyl (TEMPO), butylated hydroxytoluene (BHT), and ethene-1,1-diyldibenzene (Scheme 2B, eq a). In all cases, product formation was markedly suppressed, suggesting that a radical pathway is likely operative in this transformation. To investigate the reaction pathway, we performed a crossover experiment using two distinct ynamide-ynes (**1b** and **1n**) under standard conditions (Scheme 2B, eq b). Only the intramolecular products **3** and **15** were obtained in 62% and 47% yields, respectively; no crossover product **2bn′** was detected, indicating an intramolecular process. We then evaluated the effect of *N*-protection by testing 2-phenyl-3-(phenylethynyl)-1*H*-indole (**20**) and its *N*-tosylated analogue (**19**) under standard conditions (Scheme 2, eqs 1 and 2). The unprotected indole gave multiple products, while the *N*-tosylated compound afforded the desired product in 80% yield and short reaction time, highlighting the importance of *N*-protection.

Based on the above experimental results, control studies, and previous reports,^5,7,8^ a plausible reaction mechanism is proposed in Scheme 2C. Oxidation of Ag_2_CO_3_ by K_2_S_2_O_8_ generates Ag­(II), the sulfate radical anion (SO_4_^•–^), and the sulfate anion (SO_4_^2–^). The SO_4_^•–^ and Ag_2_CO_3_ initiate a cascade process with substrate **1**, forming intermediate **B**. The SO_4_^2–^ then abstracts the silver species from **B**, generating nitrogen anion intermediate **C** and its bond-rotated intermediate **D**. Subsequent addition and elimination steps lead to the key intermediate **E**. Two equivalents of SO_4_^•–^ then add to the rearranged alkyne to form intermediate **F**. At this stage, the S–O bond in intermediate **F** undergoes homolytic cleavage under the oxidative conditions, generating a sulfate radical and an oxygen-centered radical, which promotes the oxidative rearrangement and leads to compound **2a**.^8c^ Finally, *N*-desulfonylation occurs under the oxidative conditions (Ag_2_CO_3_/K_2_S_2_O_8_), delivering the desired product 2.^9^

## Conclusions

In conclusion, we have developed a regio- and chemoselective synthetic approach for the construction of 1,2-diketone-tethered indoles from ynamide-ynes via a skeletal rearrangement followed by oxidative *N*-desulfonylation. This protocol offers several notable advantages, including high stereospecificity, the use of an environmentally benign oxidant (K_2_S_2_O_8_) as a sustainable oxygen source, concurrent cleavage and formation of multiple bonds in a single step, scalability to gram-scale synthesis, and the potential for downstream structural elaboration.

## Supplementary Material



## Data Availability

The data underlying this study are available in the published article and its Supporting Information.

## Notes

Experimental procedures; spectral characterizations; copies of ^1^H, ^13^C {^1^H} NMR spectra for all products (PDF)
