# Supplementary material for: Ag(I)/K2S2O8‑Mediated Selective Oxidation of Ynamide-Yne via Structural Reshuffling and Consecutive N‑Desulfonylation
Source: J Org Chem. 2025 Jul 8;90(28):10031–6. doi: 10.1021/acs.joc.5c01007 (PMC12281566; doi:10.1021/acs.joc.5c01007)

Supporting Information

For

Ag(I)/$K_2S_2O_8$-Mediated Selective Oxidation of Ynamide-Yne *via* Structural Reshuffling and Consecutive *N*-Desulfonylation

Mohana Reddy Mutra,† T. L. Chandana,† Tzu-Pin Wang,† Jeh-Jeng Wang[*,†,‡]

[†]Department of Medicinal and Applied Chemistry, Kaohsiung Medical University, No. 100, Shih-Chuan 1st Rd, Sanmin District, Kaohsiung City, 807 (Taiwan).

[‡]Department of Medical Research, Kaohsiung Medical University Hospital, No. 100, Tzyou 1st Rd, Sanmin District, Kaohsiung City, 807 (Taiwan).

E-mail: jjwang@kmu.edu.tw

Table of Contents

1. **General information** S3

2. **Preparation of starting materials** S4-S5

3. **Experimental procedures** S5-S7

4. **Characterization data** S7-S11

5. **References** S12

6. **Copies of ^{1}H and ^{13}C spectra of the compounds** S13-44

1. General information

^{1}H and ^{13}C NMR spectra were recorded on a 400 MHz Varian Unity Plus or Varian Mercury plus spectrometer. The chemical shift (*δ*) values are reported in parts per million (ppm), and the coupling constants (*J*) are given in Hz. The spectra were recorded using $CDCl_3$ as a solvent. ^{1}H NMR chemical shifts are referenced to tetramethylsilane (TMS) (0 ppm). ^{13}CNMR was referenced to $CDCl_3$ (77.0 ppm). The abbreviations used are as follows: s, singlet; d, doublet; t, triplet; q, quartet; dd, doublet of doublet; ddd, doublet of doublet; dt, doublet of triplets; td, a triplet of doublet; m, multiplet; brs, broad singlet and so on. Mass spectra and high-resolution mass spectra (HRMS) were measured using the LTQ Orbitrap XL (Thermo Fisher Scientific) Liquid chromatography-mass spectrometry at National Taiwan Normal University and National Sun Yat-sen University. All commercially available reagents were used without further purification unless noted otherwise. Commercially available reagents and solvents were obtained from Sigma-Aldrich, TCI, Acros, or Alfa Aesar. Melting points were determined on an EZ-Melt (Automated melting point apparatus); the melting point number can differ (2-3) from original number due to technical error/human error. All the synthesized products showed ^{1}HNMR spectra in agreement with the assigned structures. Reaction progress and product mixtures were routinely monitored by TLC using Merck TLC aluminum sheets (silica gel 60 F254). Column chromatography was carried out with 230-400 mesh silica gel 60 (Merck) using a mixture of hexane/ethyl acetate as the eluent.

2. Preparation of Starting Materials[1]

2.1. General procedure (A) for the preparation of 4-methyl-*N*-(phenylethynyl)-*N*-(2-(phenylethynyl)phenyl)benzenesulfonamide (1a)[1]

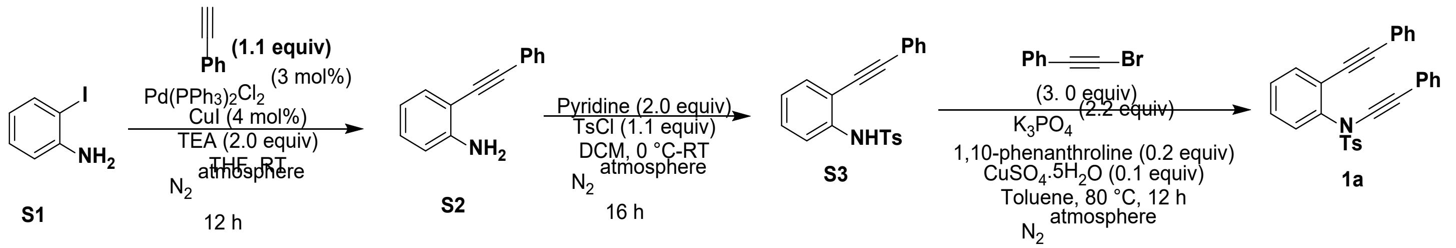

2.1a. Procedure for the synthesis of 2-(phenylethynyl)aniline (S2)

To an oven-dried Schlenk flask equipped with a magnetic stir bar was added 2-iodoaniline (10.0 g, 45.65 mmol, 1.0 equiv) in THF. The flask was evacuated and backfilled with nitrogen (three cycles). Subsequently, phenylacetylene (5.12 g, 50.22 mmol, 1.1 equiv), freshly distilled triethylamine (9.24 g, 91.31 mmol, 2.0 equiv), $Pd(PPh_3)_2Cl_2$ (0.96 g, 1.37 mmol, 3 mol%), and CuI (0.34 g, 1.82 mmol, 4 mol%) were added under a nitrogen atmosphere. The reaction mixture was stirred at room temperature for 12 h. Upon completion (monitored by TLC), the mixture was diluted with water and extracted with ethyl acetate (3 × 50 mL). The combined organic layers were dried over anhydrous Na_2SO_4, filtered, and concentrated under reduced pressure. The crude product was purified by column chromatography (silica gel, hexane/ethyl acetate = 95:5) to afford 2-(phenylethynyl)aniline as a yellow solid (7.50 g, 85% yield).

2.1b. Procedure for the synthesis of 4-methyl-*N*-(2-(phenylethynyl)phenyl)benzenesulfonamide (S3)

To an oven-dried 100 mL Schlenk flask equipped with a magnetic stir bar was added 2-(phenylethynyl)aniline (7.00 g, 36.22 mmol, 1.0 equiv) dissolved in anhydrous DCM (60 mL) and cooled to 0 °C. The flask was evacuated and backfilled with nitrogen (three cycles). Under a nitrogen atmosphere, pyridine (5.73 g, 72.45 mmol, 2.0 equiv) was added, followed by 4-methylbenzenesulfonyl chloride (TsCl) (7.59 g, 39.84 mmol, 1.1 equiv), added portionwise over 20 minutes. The reaction mixture was allowed to warm to room temperature and stirred for 16 h. If incomplete conversion was observed by TLC, the mixture was gently heated to 40 °C in an oil bath to ensure full consumption of starting material. The reaction was then cooled, and the solvent was removed under reduced pressure. The residue was dissolved in DCM, washed sequentially with water and brine, and dried over anhydrous $MgSO_4$. The crude product was purified by flash column chromatography (silica gel, hexane/ethyl acetate = 90:10) to afford 4-methyl-*N*-(2-(phenylethynyl)phenyl)benzenesulfonamide as a yellow solid (8.23 g, 63% yield).

2.1c. Procedure for the synthesis of 4-methyl-*N*-(phenylethynyl)-*N*-(2-(phenylethynyl)phenyl)benzenesulfonamide derivatives (1a)

To an oven-dried 100 mL flask equipped with a magnetic stir bar were added 4-methyl-N-(2-(phenylethynyl)phenyl)benzenesulfonamide (4.00 g, 11.52 mmol, 1.0 equiv), $CuSO_4 \cdot 5H_2O$ (288 mg, 1.15 mmol, 0.10 equiv), 1,10-phenanthroline (414 mg, 2.30 mmol, 0.20 equiv), and K_3PO_4 (5.38 g, 25.36 mmol, 2.20 equiv) in anhydrous toluene (50 mL). The flask was evacuated and refilled with nitrogen (three cycles), followed by the addition of freshly prepared bromoalkyne (6.25 g, 34.58 mmol, 3.0 equiv). The reaction mixture was stirred vigorously at 80 °C for 12 h under a nitrogen atmosphere. After cooling to room temperature, the reaction mixture was filtered through a short pad of silica gel and concentrated under reduced pressure. The crude residue was purified by flash column chromatography on silica gel (hexane/ethyl acetate = 99:1). Due to the close Rf values of the ynamide and the corresponding indole side product, a long silica column and extended elution were required. The pure product was obtained as a brown solid after washing with HPLC-grade n-pentane (3.00 g, 58% yield).

Note: Other ynamide derivatives were synthesized using the same protocol on a 300 mg scale of the corresponding S1 starting materials. Known compounds were characterized by comparison with reported ^{1}H and ^{13}C NMR data.[1]

3. Experimental procedures

3.1. General procedure (A) for the synthesis of 1-phenyl-2-(2-phenyl-1H-indol-3-yl)ethane-1,2-dione (2-25)

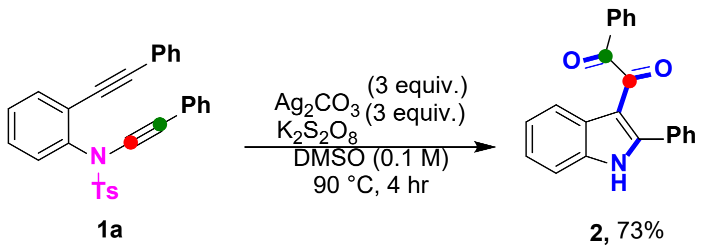

A 15 mL oven-dried sealed glass tube equipped with a magnetic stir bar was charged with 4-methyl-N-(phenylethynyl)-N-(2-(phenylethynyl)phenyl)benzenesulfonamide (68 mg, 0.15 mmol, 1.0 equiv), Ag_2CO_3 (125 mg, 0.45 mmol, 3.0 equiv), $K_2S_2O_8$ (123 mg, 0.45 mmol, 3.0 equiv), and dimethyl sulfoxide (DMSO) (0.1 M). The reaction mixture was sealed and heated at 90 °C for 4 hr with stirring (reaction monitored by TLC). Upon completion, the mixture was cooled to room temperature, diluted with water (10 mL), and extracted with ethyl acetate (3 × 15 mL). The combined organic layers were washed with brine, dried over anhydrous Na_2SO_4, filtered, and concentrated under reduced pressure using a rotary evaporator. The crude product was purified by

flash column chromatography on silica gel (hexane/ethyl acetate = 86:14) to afford 1-phenyl-2-(2-phenyl-1H-indol-3-yl)ethane-1,2-dione as an yellow solid (xx mg, 73% yield).

3.2. Gram-scale synthesis of 1-phenyl-2-(2-phenyl-1H-indol-3-yl)ethane-1,2-dione (2)

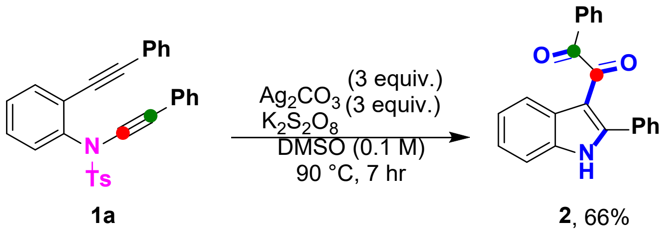

A 50 mL oven-dried sealed glass tube equipped with a magnetic stir bar was charged with 4-methyl-N-(phenylethynyl)-*N*-(2-(phenylethynyl)phenyl)benzenesulfonamide (1.0 g, 2.23 mmol, 1.0 equiv), Ag_2CO_3 (1.83 g, 6.69 mmol, 3.0 equiv), $K_2S_2O_8$ (1.80 g, 6.69 mmol, 3.0 equiv), and dimethyl sulfoxide (DMSO) (0.1 M). The reaction mixture was sealed and heated at 90 °C for 7 hr with stirring (reaction monitored by TLC). Upon completion, the mixture was cooled to room temperature, diluted with water (50 mL), and extracted with ethyl acetate (3 × 50 mL). The combined organic layers were washed with brine, dried over anhydrous Na_2SO_4, filtered, and concentrated under reduced pressure using a rotary evaporator. The crude product was purified by flash column chromatography on silica gel (hexane/ethyl acetate = 85:15) to afford 1-phenyl-2-(2-phenyl-1H-indol-3-yl)ethane-1,2-dione as an yellow solid (xx mg, 66% yield).

3.3. Synthesis of 1-phenyl-2-(2-phenyl-1H-indol-3-yl)ethane-1,2-diol (x)

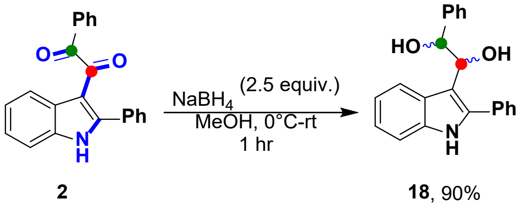

In an oven-dried 15 mL Schlenk tube equipped with a magnetic stir bar, 1-phenyl-2-(2-phenyl-1H-indol-3-yl)ethane-1,2-dione (100 mg, 0.30 mmol, 1.0 equiv) was dissolved in anhydrous methanol (4 mL). The solution was cooled to 0 °C, and sodium borohydride (29 mg, 0.75 mmol, 2.5 equiv) was added slowly in small portions. The resulting mixture was allowed to warm to room temperature and stirred for 1 hr. The progress of the reaction was monitored by TLC. Upon completion, the reaction mixture was concentrated under reduced pressure using a rotary evaporator. The crude product was purified by flash column chromatography on silica gel (hexane/ethyl acetate = 75:25) to afford 1-phenyl-2-(2-phenyl-1H-indol-3-yl)ethane-1,2-diol as a white solid (91 mg, 90% yield).

3.4. Synthesis of 1-phenyl-2-(2-phenyl-1-tosyl-indol-3-yl)ethane-1,2-dione (2a)

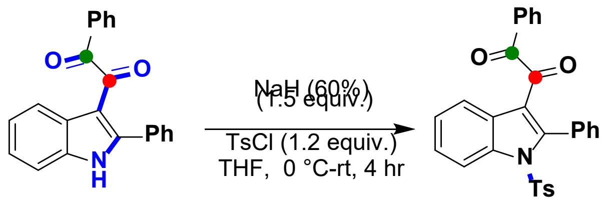

2 **2a**, 65%

An oven-dried round bottom flask equipped with a magnetic stir bar was charged with 1-phenyl-2-(2-phenyl-1H-indol-3-yl)ethane-1,2-dione (100 mg, 0.30 mmol, 1.0 equiv) in anhydrous THF (3 mL) and cooled to 0 °C. To the reaction mixture, sodium hydride (60% dispersion in mineral oil, 18 mg, 0.45 mmol, 1.5 equiv) and 4-toluenesulfonyl chloride (68 mg, 0.36 mmol, 1.2 equiv) were added portion-wise under a nitrogen atmosphere. The reaction mixture was stirred at room temperature for 4 h. Upon completion (monitored by TLC), the mixture was quenched with an aqueous solution of NH_4Cl (30 mL). The organic layer was separated, and the aqueous phase was extracted with ethyl acetate (3 × 20 mL). The combined organic extracts were washed with water (30 mL), followed by brine (30 mL), dried over anhydrous Na_2SO_4, filtered, and concentrated under reduced pressure. Purification of the crude product by flash column chromatography on silica gel (hexane/ethyl acetate = 90:10) afforded 1-phenyl-2-(2-phenyl-1-tosyl-indol-3-yl)ethane-1,2-dione as a yellow solid (96 mg, 65% yield).

4. Characterization data

1-phenyl-2-(2-phenyl-1H-indol-3-yl)ethane-1,2-dione (2):[2] The title compound was prepared according to the

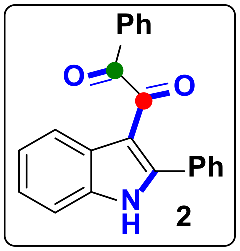

procedure described in Section 4.1. Purification by column chromatography on silica gel (hexane/ethyl acetate = 86:14) afforded the product as a yellow solid (36 mg, 73% yield); **mp** 201–203 °C. **^{1}H NMR** (400 MHz, DMSO-d_6) δ 12.63 (brs, 1H), 8.17 (d, J = 7.8 Hz, 1H), 7.70 – 7.64 (m, 3H), 7.53 (d, J = 7.7 Hz, 1H), 7.50-7.46 (m, 2H), 7.40 – 7.28 (m, 5H), 7.23-7.19 (m, 2H). **^{13}C{^{1}H} NMR** (101 MHz, DMSO-d_6) δ 193.6, 190.3, 148.7, 136.0, 134.2, 133.1, 130.5, 130.1, 129.7, 129.2, 128.8, 127.9, 126.8, 123.8, 122.9, 121.0, 112.3, 110.2. **HRMS** (ESI) calcd for $C_{22}H_{15}O_2NNa$ [M+Na]$^+$ 348.0995; found: 348.0992.

1-phenyl-2-(2-phenyl-1-tosyl-indol-3-yl)ethane-1,2-dione (2a): The title compound was prepared according

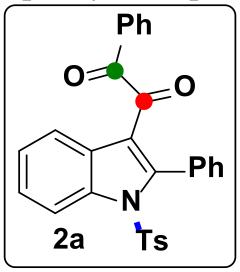

to the procedure described in Section 4.4. Purification by column chromatography on silica gel (hexane/ethyl acetate = 90:10) afforded the product as a yellow solid (96 mg, 65% yield); **mp** 174–175 °C. **^{1}H NMR** (399 MHz, $CDCl_3$) δ 8.47 – 8.43 (m, 1H), 8.42 – 8.38 (m, 1H), 7.55 – 7.45 (m, 5H), 7.33 (d, J = 8.4 Hz, 2H), 7.30 – 7.25 (m, 2H ($CDCl_3$ solvlent peak merged; therefore 3 H were shown in spectra), 7.19 (ddt, J = 8.8, 6.8, 2.0 Hz, 1H), 7.12 (dd, J = 8.6, 0.6 Hz, 2H), 7.01 – 6.91 (m, 4H), 2.35 (s, 3H). **^{13}C{^{1}H} NMR** (100 MHz, $CDCl_3$) δ 192.3, 191.7, 147.9, 145.7, 136.5, 135.4, 133.9, 132.7, 132.5, 130.1, 129.7, 129.3, 128.1, 127.9, 127.1, 126.9, 126.7,

126.4, 125.5, 122.4, 119.0, 115.0, 21.6. **HRMS** (ESI) calcd for $C_{29}H_{21}O_4NSNa$ $[M+Na]^+$ 502.1083; found: 502.1080.

1-(5-methyl-2-phenyl-1H-indol-3-yl)-2-phenylethane-1,2-dione (3): The title compound was prepared according to the procedure described in Section 4.1. Purification by column chromatography on silica gel (hexane/ethyl acetate = 86:14) afforded the product as a yellow solid (36 mg, 70% yield); **mp** 179–180 °C. **^{1}H NMR** (399 MHz, $CDCl_3$) δ 8.85 (brs, 1H), 8.25 (s, 1H), 7.68 (dd, J = 8.4, 1.3 Hz, 2H), 7.53 – 7.48 (m, 1H), 7.35 – 7.24 (m, 4H), 7.20 (dd, J = 8.3, 1.3 Hz, 2H), 7.15 (dd, J = 8.3, 1.2 Hz, 1H), 7.11 – 7.06 (m, 2H), 2.50 (s, 3H). **^{13}C{^{1}H} NMR** (100 MHz, $CDCl_3$) δ 193.8, 191.1, 148.1, 133.7, 133.7, 133.2, 130.5, 129.9, 129.8, 129.5, 128.4, 128.1, 127.5, 125.8, 122.2, 111.5, 110.8, 21.7. **HRMS** (ESI) calcd for $C_{23}H_{17}O_2NNa$ $[M+Na]^+$ 362.1151; found: 362.1148.

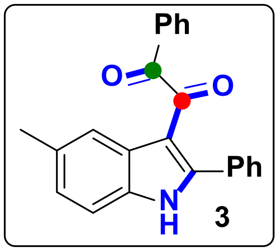

1-(5-ethyl-2-phenyl-1H-indol-3-yl)-2-phenylethane-1,2-dione (4): The title compound was prepared according to the procedure described in Section 4.1. Purification by column chromatography on silica gel (hexane/ethyl acetate = 85:15) afforded the product as a yellow solid (35 mg, 65% yield); **mp** 173–174 °C. **^{1}H NMR** (399 MHz, $CDCl_3$) δ 8.65 (brs, 1H), 8.30 (s, 1H), 7.70 (dd, J = 8.4, 1.3 Hz, 2H), 7.54 – 7.49 (m, 1H), 7.35 – 7.27 (m, 4H), 7.25 – 7.19 (m, 3H), 7.16 – 7.11 (m, 2H), 2.82 (q, J = 7.6 Hz, 2H), 1.32 (t, J = 7.6 Hz, 3H). **^{13}C{^{1}H} NMR** (100 MHz, $CDCl_3$) δ 193.7, 191.1, 148.0, 140.0, 133.8, 133.7, 130.6, 130.0, 129.8, 129.5, 128.4, 128.1, 127.5, 124.9, 121.2, 111.8, 110.8, 29.2, 16.5. **HRMS** (ESI) calcd for $C_{24}H_{19}O_2NNa$ $[M+Na]^+$ 376.1308; found: 376.1304.

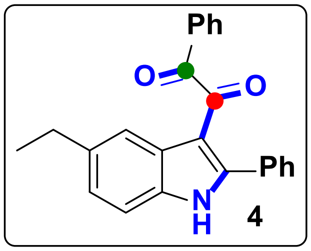

1-(5-chloro-2-phenyl-1H-indol-3-yl)-2-phenylethane-1,2-dione (5): The title compound was prepared according to the procedure described in Section 4.1. Purification by column chromatography on silica gel (hexane/ethyl acetate = 85:15) afforded the product as yellow solid (37 mg, 68% yield). **^{1}H NMR** (400 MHz, $CDCl_3$+3 drops of DMSO-d_6) 11.54 (brs, 1H), 8.42 (d, J = 2.0 Hz, 1H), 7.67 (dd, J = 8.3, 1.1 Hz, 2H), 7.55 – 7.50 (m, 1H), 7.40 – 7.29 (m, 4H), 7.28 – 7.23 (m, 3H), 7.13 (t, J = 7.7 Hz, 2H). **^{13}C{^{1}H} NMR** (100 MHz, $CDCl_3$+3 drops of DMSO-d_6) δ 193.6, 190.6, 149.4, 134.4, 133.7, 133.4, 130.4, 130.1, 129.6, 129.3, 128.6, 128.3, 128.2, 127.8, 124.1, 121.5, 112.8, 110.9. **HRMS** (ESI) calcd for $C_{22}H_{14}O_2NClNa$ $[M+Na]^+$ 382.0605; found: 382.0602.

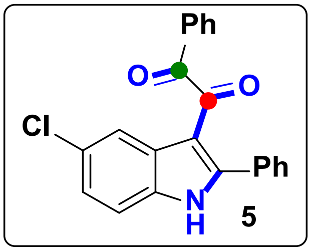

1-phenyl-2-(2-(o-tolyl)-1H-indol-3-yl)ethane-1,2-dione (6): The title compound was prepared according to the procedure described in Section 4.1. Purification by column chromatography on silica gel (hexane/ethyl acetate = 86:14) afforded the product as a yellow solid (26 mg, 50% yield); **mp** 213–215 °C. **^{1}H NMR** (399 MHz, $CDCl_3$) δ 8.73 (brs, 1H), 8.50 (d, J = 8.3 Hz, 1H), 7.52 (dd, J = 8.4, 1.3 Hz, 2H), 7.50 – 7.45 (m, 1H), 7.42 – 7.32 (m, 3H),

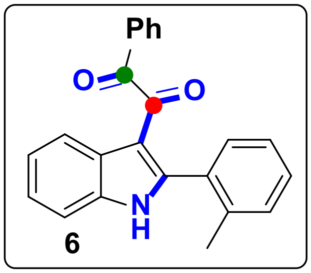

7.29 – 7.24 (m, 2H), 7.16 (td, J = 7.6, 1.4 Hz, 1H), 7.00 (d, J = 7.7 Hz, 1H), 6.93 (dd, J = 7.6, 1.2 Hz, 1H), 6.81 (t, J = 7.5 Hz, 1H), 1.99 (s, 3H). **^{13}C{^{1}H} NMR** (100 MHz, $CDCl_3$) δ 193.8, 191.3, 147.7, 138.2, 135.3, 133.7, 133.2, 131.4, 130.0, 129.7, 129.3, 129.1, 128.2, 126.7, 124.9, 124.3, 123.4, 122.6, 112.8, 111.1, 19.7. **HRMS** (ESI) calcd for $C_{23}H_{17}O_2NNa$ $[M+Na]^+$ 362.1151; found: 362.1149.

1-phenyl-2-(2-(p-tolyl)-1H-indol-3-yl)ethane-1,2-dione (7): The title compound was prepared according to the procedure described in Section 4.1. Purification by column chromatography on silica gel (hexane/ethyl acetate = 86:14) afforded the product as a yellow solid (39 mg, 75% yield); **mp** 207–208 °C. **^{1}H NMR** (399 MHz, $CDCl_3$) δ 9.04 (brs, 1H), 8.36 (dd, J = 8.0, 1.2 Hz, 1H), 7.60 (d, J = 8.2 Hz, 2H), 7.38 – 7.26 (m, 4H), 7.25 – 7.21 (m, 2H), 7.11 (dddd, J = 9.5, 7.6, 2.9, 1.0 Hz, 4H), 2.38 (s, 3H). **^{13}C{^{1}H} NMR** (100 MHz, $CDCl_3$) δ 193.5, 191.2, 147.9, 144.9, 135.4, 131.2, 130.5, 129.9, 129.8, 129.7, 129.2, 128.1, 127.2, 124.2, 123.4, 122.3, 111.7, 111.3, 21.8. **HRMS** (ESI) calcd for $C_{23}H_{17}O_2NNa$ $[M+Na]^+$ 362.1151; found: 362.1149.

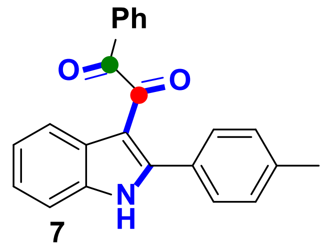

7

1-(2-(4-ethylphenyl)-1H-indol-3-yl)-2-phenylethane-1,2-dione (8): The title compound was prepared according to the procedure described in Section 4.1. Purification by column chromatography on silica gel (hexane/ethyl acetate = 87:13) afforded the product as a yellow solid (36 mg, 69% yield); **mp** 199–200 °C. **^{1}H NMR** (399 MHz, $CDCl_3$) δ 8.84 (brs, 1H), 8.44 – 8.37 (m, 1H), 7.69 (dd, J = 8.4, 1.3 Hz, 2H), 7.53 – 7.48 (m, 1H), 7.41 – 7.29 (m, 5H), 7.14 (d, J = 8.3 Hz, 2H), 6.92 (d, J = 8.4 Hz, 2H), 2.57 (q, J = 7.6 Hz, 2H), 1.18 (t, J = 7.6 Hz, 3H). **^{13}C{^{1}H} NMR** (100 MHz, $CDCl_3$) δ 193.9, 191.1, 148.3, 146.4, 135.4, 133.7, 133.6, 130.0, 129.5, 128.3, 127.7, 127.6, 127.3, 124.2, 123.4, 122.4, 111.8, 111.1, 28.7, 15.4. HRMS (ESI) calcd for $C_{24}H_{19}O_2NNa$ $[M+Na]^+$ 376.1308; found: 376.1305.

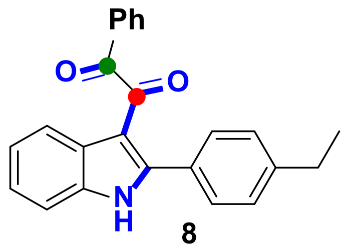

8

1-(2-(4-methoxyphenyl)-1H-indol-3-yl)-2-phenylethane-1,2-dione (9): The title compound was prepared according to the procedure described in Section 4.1. Purification by column chromatography on silica gel (hexane/ethyl acetate = 84:16) afforded the product as a yellow solid (32 mg, 61% yield); **mp** 161–162 °C. **^{1}H NMR** (399 MHz, $CDCl_3$) δ 8.80 (brs, 1H), 8.38 (dd, J = 7.8, 1.4 Hz, 1H), 7.73 (dd, J = 8.4, 1.3 Hz, 2H), 7.55 – 7.50 (m, 1H), 7.40 – 7.29 (m, 5H), 7.18 (d, J = 8.8 Hz, 2H), 6.62 (d, J = 8.8 Hz, 2H), 3.75 (s, 3H). **^{13}C{^{1}H} NMR** (100 MHz, $CDCl_3$) δ 194.0, 191.0, 160.9, 148.1, 135.4, 133.9, 133.7, 131.4, 129.5, 128.4, 127.3, 124.2, 123.4, 122.5, 122.3, 113.7, 111.1, 55.3. HRMS (ESI) calcd for $C_{23}H_{17}O_3NNa$ $[M+Na]^+$ 378.1100; found: 378.1099.

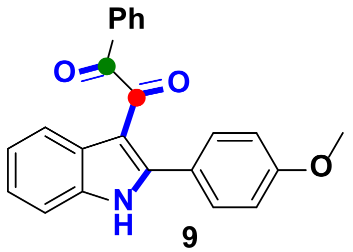

9

1-(2-(3-chlorophenyl)-1H-indol-3-yl)-2-phenylethane-1,2-dione (10): The title compound was prepared

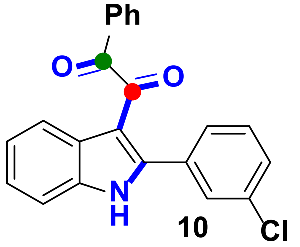

10

according to the procedure described in Section 4.1. Purification by column chromatography on silica gel (hexane/ethyl acetate = 85:15) afforded the product as a yellow solid (31 mg, 56% yield); **mp** 194–195 °C. **^{1}H NMR** (399 MHz, $CDCl_3$) δ 8.85 (brs, 1H), 8.38 (dd, J = 5.6, 3.6 Hz, 1H), 7.76 (dd, J = 8.3, 1.3 Hz, 2H), 7.58 – 7.53 (m, 1H), 7.44 – 7.33 (m, 5H), 7.30 – 7.26 (m, 1H), 7.20 (dd, J = 7.3, 1.4 Hz, 2H), 7.11 (t, J = 8.0 Hz, 1H). **^{13}C{^{1}H} NMR** (100 MHz, $CDCl_3$) δ 193.6, 190.8, 145.7, 135.4, 134.4, 134.1, 133.4, 132.2, 130.0, 129.9, 129.5, 128.7, 128.1, 127.1, 124.7, 123.7, 122.5, 112.1, 111.2. **HRMS** (ESI) calcd for $C_{22}H_{14}O_2NClNa$ $[M+Na]^+$ 382.0605; found: 382.0604.

methyl 4-(3-(2-oxo-2-phenylacetyl)-1H-indol-2-yl)benzoate (11): The title compound was prepared according

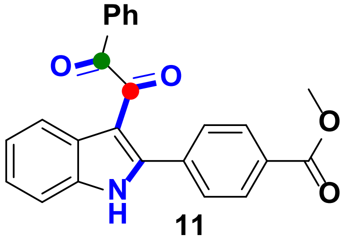

11

to the procedure described in Section 4.1. Purification by column chromatography on silica gel (hexane/ethyl acetate = 80:20) afforded the product as a pale-yellow solid (38 mg, 66% yield); **mp** 187–188 °C. **^{1}H NMR** (399 MHz, $CDCl_3$) δ 9.37 (brs, 1H), 8.30 (d, J = 9.2 Hz, 1H), 7.75 – 7.67 (m, 4H), 7.56 – 7.51 (m, 1H), 7.42 – 7.29 (m, 7H), 3.90 (s, 3H). **^{13}C{^{1}H} NMR** (100 MHz, $CDCl_3$) δ 193.8, 190.7, 166.4, 146.2, 135.6, 134.9, 134.2, 133.4, 131.0, 129.9, 129.6, 129.2, 128.6, 127.1, 124.6, 123.6, 122.3, 111.9, 111.5, 52.4. **HRMS** (ESI) calcd for $C_{24}H_{17}O_4NNa$ $[M+Na]^+$ 406.1049; found: 406.1046.

1-(4-methoxyphenyl)-2-(2-phenyl-1H-indol-3-yl)ethane-1,2-dione (12): The title compound was prepared

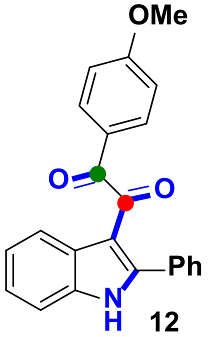

12

according to the procedure described in Section 4.1. Purification by column chromatography on silica gel (hexane/ethyl acetate = 85:15) afforded the product as a yellow solid (33 mg, 62% yield); **mp** 169–170 °C. **^{1}H NMR** (399 MHz, $CDCl_3$) δ 8.97 (brs, 1H), 8.37 (d, J = 8.4 Hz, 1H), 7.73 (dd, J = 8.3, 1.2 Hz, 2H), 7.55 – 7.49 (m, 1H), 7.38 – 7.27 (m, 5H), 7.14 (d, J = 8.8 Hz, 2H), 6.57 (d, J = 8.8 Hz, 2H), 3.72 (s, 3H). **^{13}C{^{1}H} NMR** (100 MHz, $CDCl_3$) δ 194.0, 191.0, 160.8, 148.2, 135.4, 133.8, 133.6, 131.3, 129.5, 128.4, 127.3, 124.2, 123.3, 122.5, 122.2, 113.6, 111.6, 111.2, 55.3. HRMS (ESI) calcd for $C_{23}H_{17}O_3NNa$ $[M+Na]^+$ 378.1100; found: 378.1099.

1-(3-chlorophenyl)-2-(2-phenyl-1H-indol-3-yl)ethane-1,2-dione (13): The title compound was prepared

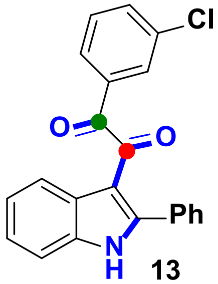

13

according to the procedure described in Section 4.1. Purification by column chromatography on silica gel (hexane/ethyl acetate = 87:13) afforded the product as a yellow solid (37 mg, 67% yield); **mp** 188–189 °C. **^{1}H NMR** (399 MHz, $CDCl_3$) δ 8.81 (brs, 1H), 8.41 (dd, J = 7.7, 1.5 Hz, 1H), 7.64 – 7.56 (m, 2H), 7.51 – 7.47 (m, 1H), 7.44 – 7.32 (m, 4H), 7.29 (d, J = 7.8 Hz, 1H), 7.26 – 7.21 (m, 2H), 7.20 – 7.15 (m, 2H). **^{13}C{^{1}H} NMR** (100 MHz, $CDCl_3$) δ 192.3, 190.1, 148.1, 135.4, 135.2, 134.8, 133.7, 130.3, 130.1,

130.0, 129.7, 129.0, 128.3, 127.7, 127.1, 124.6, 123.6, 122.4, 111.8, 111.2. **HRMS** (ESI) calcd for $C_{22}H_{14}O_2NClNa$ $[M+Na]^+$ 382.0605; found: 382.0603.

1-(3-nitrophenyl)-2-(2-phenyl-1H-indol-3-yl)ethane-1,2-dione (14): The title compound was prepared according to the procedure described in Section 4.1. Purification by column chromatography on silica gel (hexane/ethyl acetate = 80:20) afforded the product as a yellow solid (23 mg, 41% yield); **mp** 227–228 °C. **^{1}H NMR** (399 MHz, $CDCl_3$) δ 8.74 (s, 1H), 8.49 – 8.42 (m, 2H), 8.37 (ddd, J = 8.2, 2.3, 1.1 Hz, 1H), 8.05 (ddd, J = 7.7, 1.5, 1.1 Hz, 1H), 7.59 – 7.54 (m, 1H), 7.48 – 7.32 (m, 4H), 7.28 (d, J = 1.4 Hz, 1H), 7.25 (d, J = 1.5 Hz, 1H), 7.22 – 7.15 (m, 2H). **^{13}C{^{1}H} NMR** (100 MHz, $CDCl_3$) δ 191.0, 189.3, 148.2, 135.4, 135.1, 134.8, 130.3, 130.0, 129.6, 128.4, 127.8, 127.1, 124.8, 123.9, 123.9, 122.5, 112.0, 111.2. **HRMS** (ESI) calcd for $C_{22}H_{14}O_4N_2Na$ $[M+Na]^+$ 393.0845; found: 393.0842.

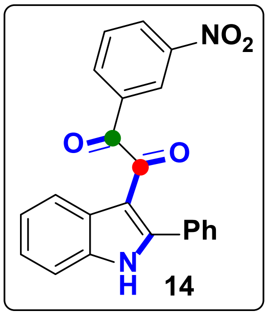

methyl 4-(3-(2-oxo-2-phenylacetyl)-1H-indol-2-yl)benzoate (15): The title compound was prepared according to the procedure described in Section 4.1. Purification by column chromatography on silica gel (hexane/ethyl acetate = 80:20) afforded the product as a pale-yellow solid (31 mg, 54% yield); **mp** 218–219 °C. **^{1}H NMR** (399 MHz, $CDCl_3$) δ 8.97 (brs, 1H), 8.42 (dd, J = 7.8, 1.4 Hz, 1H), 7.98 (d, J = 8.7 Hz, 2H), 7.74 (d, J = 8.7 Hz, 2H), 7.39 (dddd, J = 11.6, 6.7, 2.9, 1.2 Hz, 3H), 7.30 (ddd, J = 13.6, 6.7, 1.5 Hz, 1H), 7.21 (dd, J = 8.3, 1.3 Hz, 2H), 7.14 – 7.08 (m, 2H), 3.94 (s, 3H). **^{13}C{^{1}H} NMR** (100 MHz, $CDCl_3$) δ 192.9, 190.3, 166.2, 148.2, 136.8, 135.5, 134.2, 130.3, 130.1, 130.0, 129.5, 129.3, 128.3, 127.1, 124.6, 123.6, 122.4, 111.8, 111.3, 110.0, 52.5. **HRMS** (ESI) calcd for $C_{24}H_{17}O_4NNa$ $[M+Na]^+$ 406.1049; found: 406.1049.

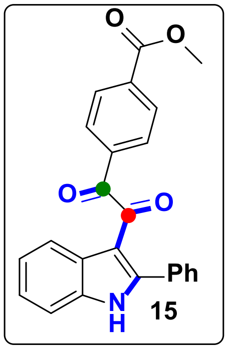

1-phenyl-2-(2-phenyl-1H-indol-3-yl)ethane-1,2-diol (18): The title compound was prepared according to the procedure described in Section 4.3. Purification by column chromatography on silica gel (hexane/ethyl acetate = 75:25) afforded the products (diastereomers) a white solid (91 mg, 90% yield); **mp** 138–139 °C. **^{1}H NMR** (400 MHz, DMSO-d_6) δ 11.15 (s, 1H), 7.91 (d, J = 8.0 Hz, 1H), 7.86 – 7.81 (m, 2H), 7.55 – 7.45 (m, 4H), 7.43 – 7.29 (m, 4H), 7.27 – 7.21 (m, 1H), 7.12 – 7.07 (m, 1H), 6.99 (ddd, J = 8.0, 7.0, 1.0 Hz, 1H), 5.21 (d, J = 3.9 Hz, 1H), 5.01 (dd, J = 7.7, 3.9 Hz, 1H), 4.85 (d, J = 3.2 Hz, 1H), 4.82 (d, J = 3.8 Hz, 1H). **^{13}C{^{1}H} NMR** (101 MHz, DMSO-d_6) δ 145.3, 136.3, 136.1, 133.1, 129.0, 128.3, 127.5, 127.5, 127.2, 126.7, 121.5, 121.1, 118.3, 114.5, 111.1, 75.8, 71.0. **HRMS** (ESI) calcd for $C_{22}H_{19}O_2NNa$ $[M+Na]^+$ 352.1308; found: 352.1304.

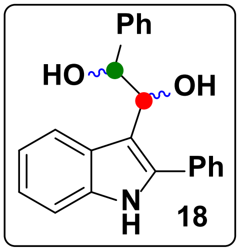

5. References

1) Mutra, M. R.; Wang, J.-J. Photoinduced ynamide structural reshuffling and functionalization. *Nat. Commun*. **2022**, 13, 2345.

2) a) Zhou, J.; Li, J.; Li, Y.; Wu, C.; He, G.; Yang, Q.; Zhou, Y.; Liu, H. Direct Synthesis of 3-Acylindoles through Rhodium(III)-Catalyzed Annulation of N-Phenylamidines with α-Chloro Ketones. *Org. Lett*. **2018**, 20, 7645–7649; b) Tang, R.-Y.; Guo, X.-K.; Xiang, J.-N.; Li, J.-H. Palladium-Catalyzed Synthesis of 3-Acylated Indoles Involving Oxidative Cross-Coupling of Indoles with α-Amino Carbonyl Compounds. *J. Org. Chem*. **2013**, 78, 11163–11171; c) b) Mutra, M. R.; Chandana, T. L.; Wang, J.-J. Atom-economical and workup-free multiparticipation of *p*-TsOH in yne-ynamide skeletal reshuffle: access to regiospecific, chemospecific, and stereospecific (*E*)-alkenyl sulfonate/ketone-tethered indoles. *Green Chem*. **2025**, 27, 1062–1072.

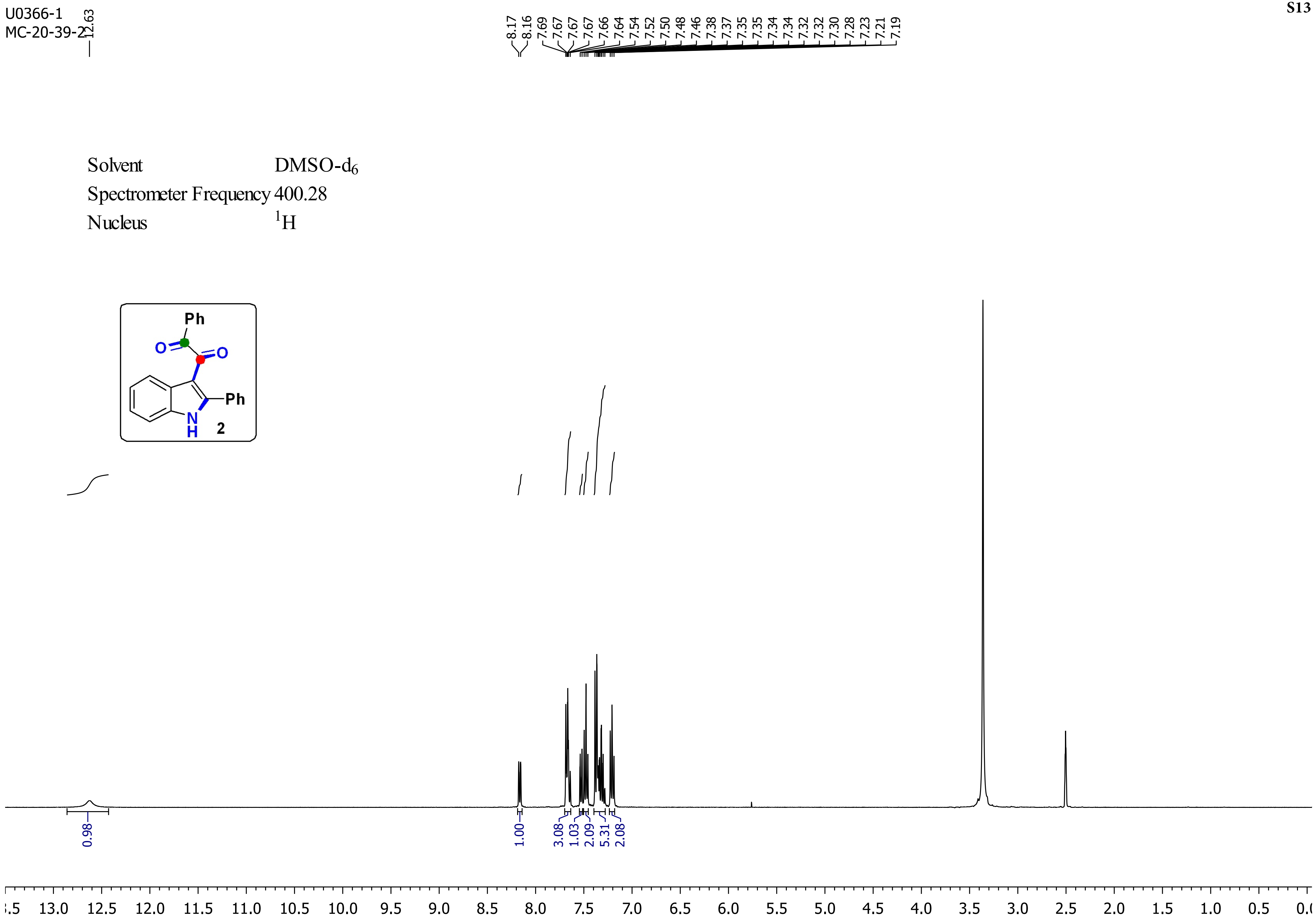
U0366-1
MC-20-39-2
12.63
8.17
8.16
7.69
7.67
7.67
7.66
7.64
7.54
7.52
7.50
7.48
7.46
7.38
7.37
7.35
7.35
7.34
7.34
7.32
7.32
7.30
7.28
7.23
7.21
7.19
Solvent DMSO-d6
Spectrometer Frequency 400.28
Nucleus 1H
Ph
O
O
Ph
N
H
2
0.98
1.00
3.08
1.03
2.09
5.31
2.08
13.0 12.5 12.0 11.5 11.0 10.5 10.0 9.5 9.0 8.5 8.0 7.5 7.0 6.5 6.0 5.5 5.0 4.5 4.0 3.5 3.0 2.5 2.0 1.5 1.0 0.5

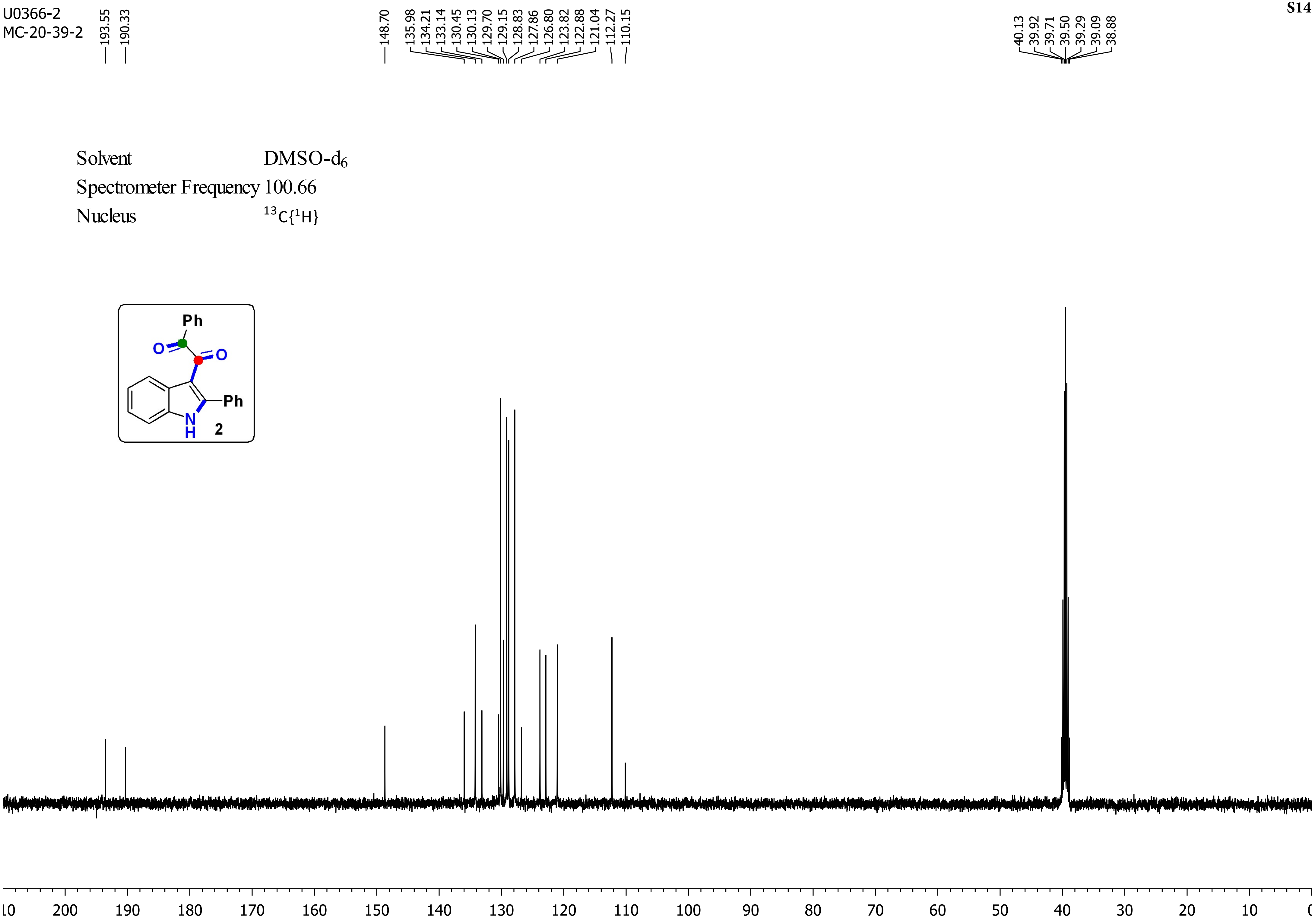
U0366-2
MC-20-39-2
193.55
190.33
148.70
135.98
134.21
133.14
130.45
130.13
129.70
129.15
128.83
127.86
126.80
123.82
122.88
121.04
112.27
110.15
40.13
39.92
39.71
39.50
39.29
39.09
38.88
Solvent DMSO-d6
Spectrometer Frequency 100.66
Nucleus 13C{1H}
Ph
O
O
Ph
N
H
2
200
190
180
170
160
150
140
130
120
110
100
90
80
70
60
50
40
30
20
10

U0185-5
MC-21-17

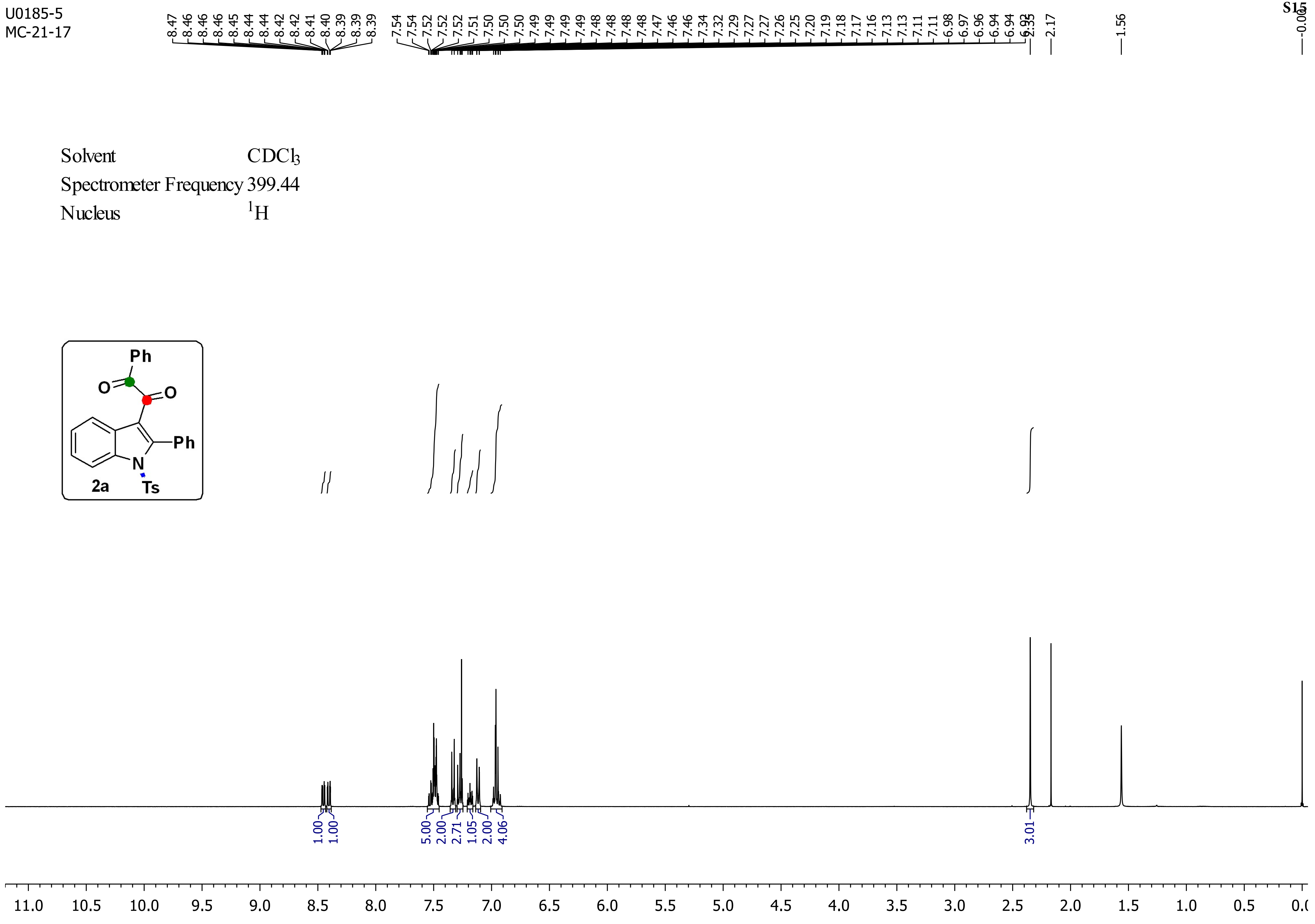

U0185-6
MC-21-17

Solvent CDCl$_3$
Spectrometer Frequency 100.45
Nucleus $^{13}C\{^{1}H\}$

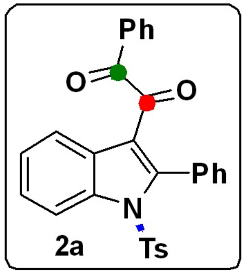

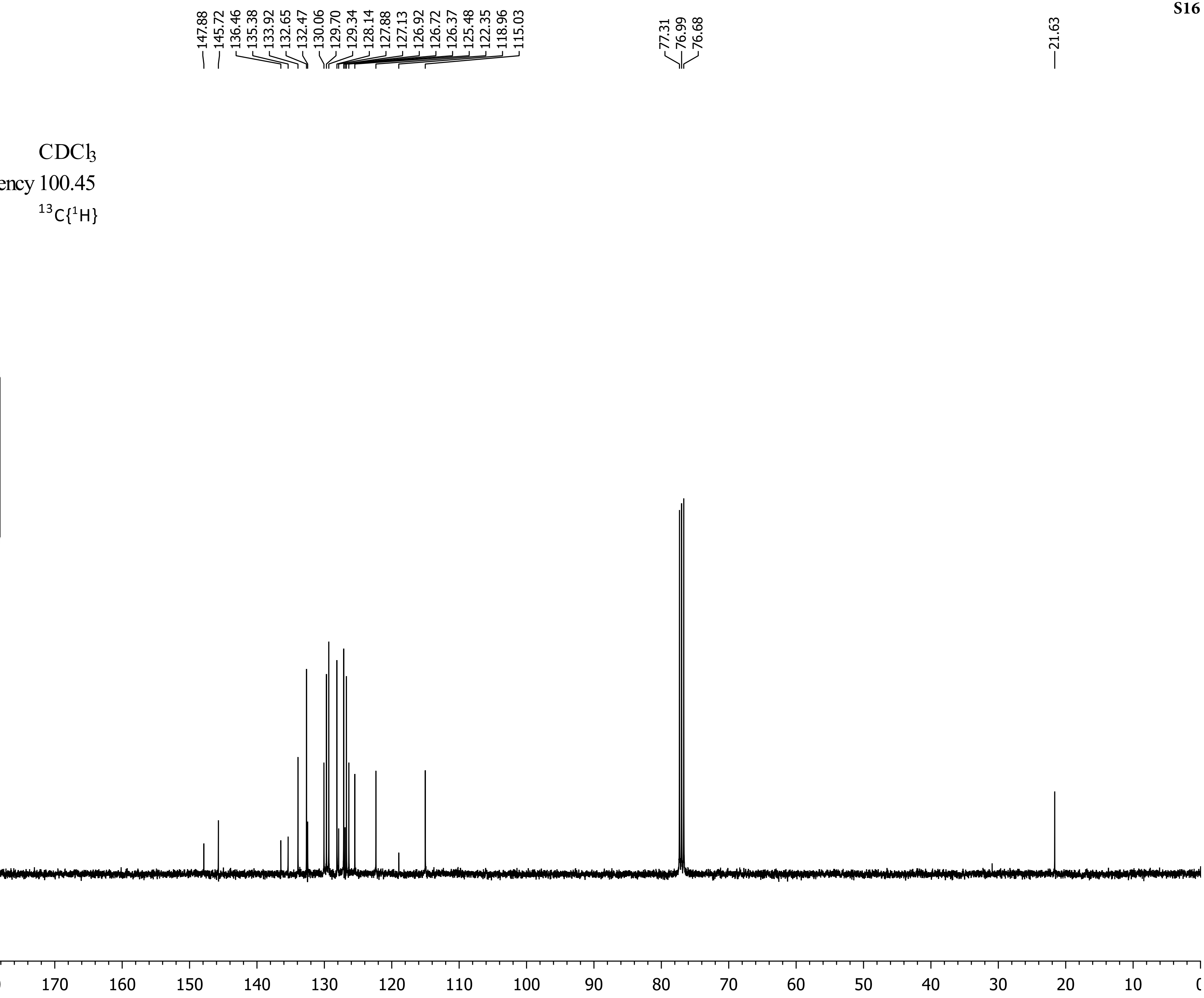

U0135-3
MC-21-6

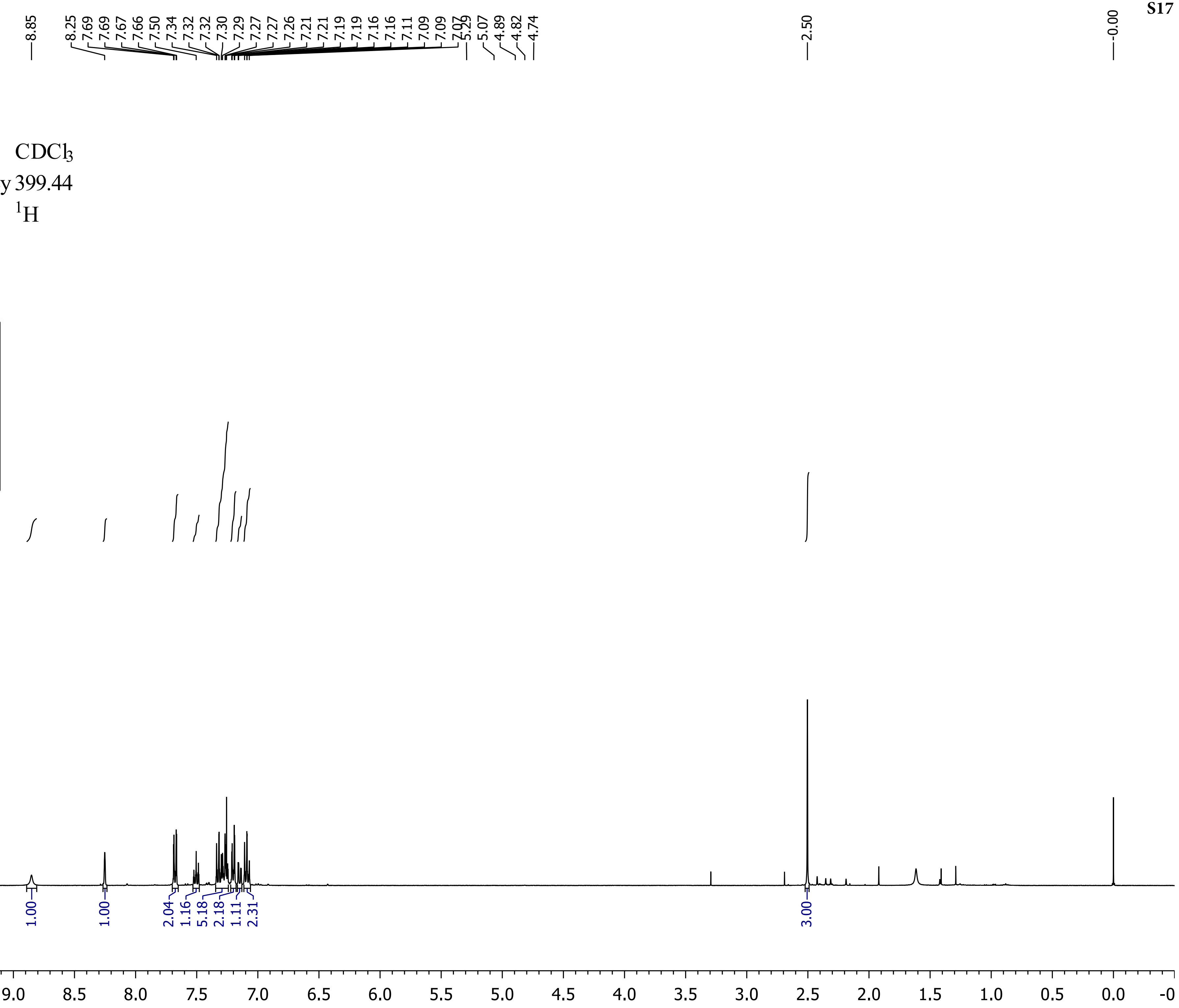

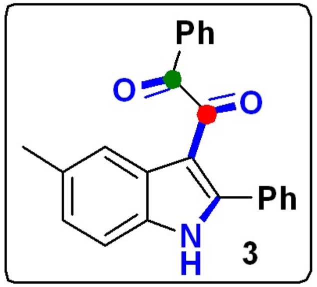

U0135-4
MC-21-6

193.79, 191.07, 148.07, 133.71, 133.66, 133.22, 130.50, 129.94, 129.77, 129.48, 128.36, 128.08, 127.45, 125.83, 122.17, 111.53, 110.82, 77.31, 76.99, 76.67, 21.65

Solvent	$CDCl_3$
Spectrometer Frequency	100.45
Nucleus	$^{13}C\{^1H\}$

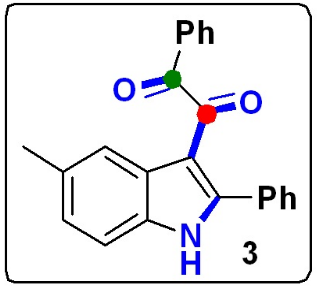

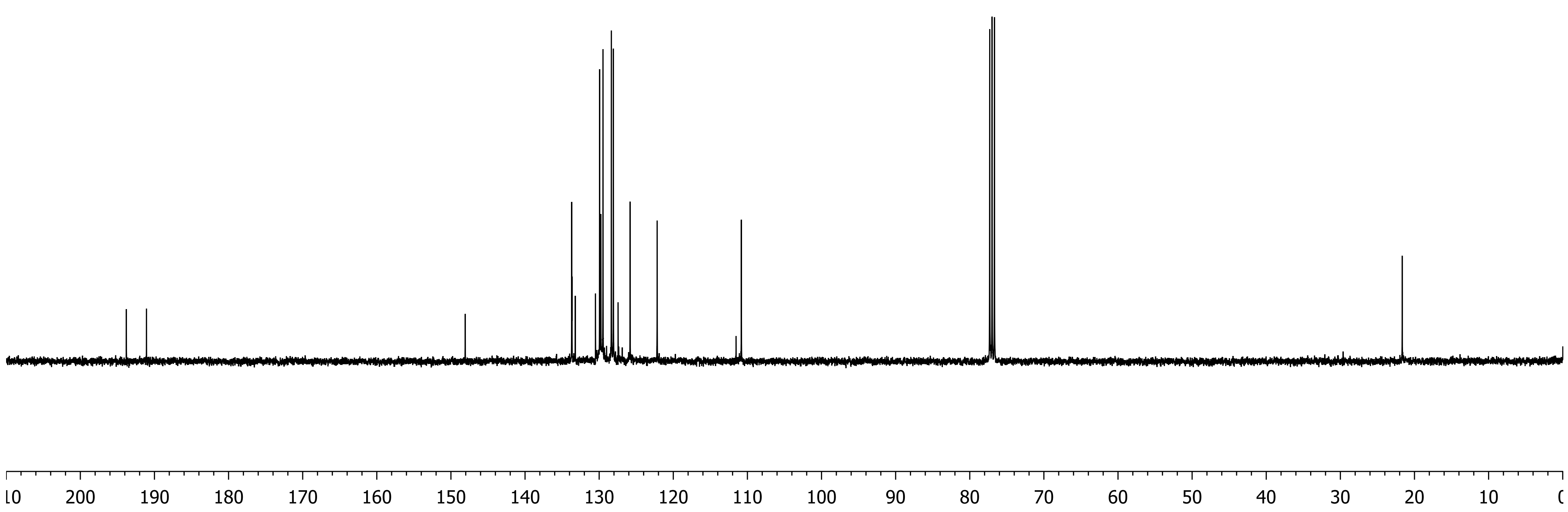

U0146-1
MC-21-10

Solvent CDCl$_3$
Spectrometer Frequency 399.44
Nucleus ^{1}H

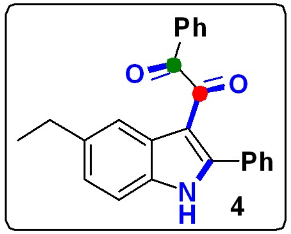

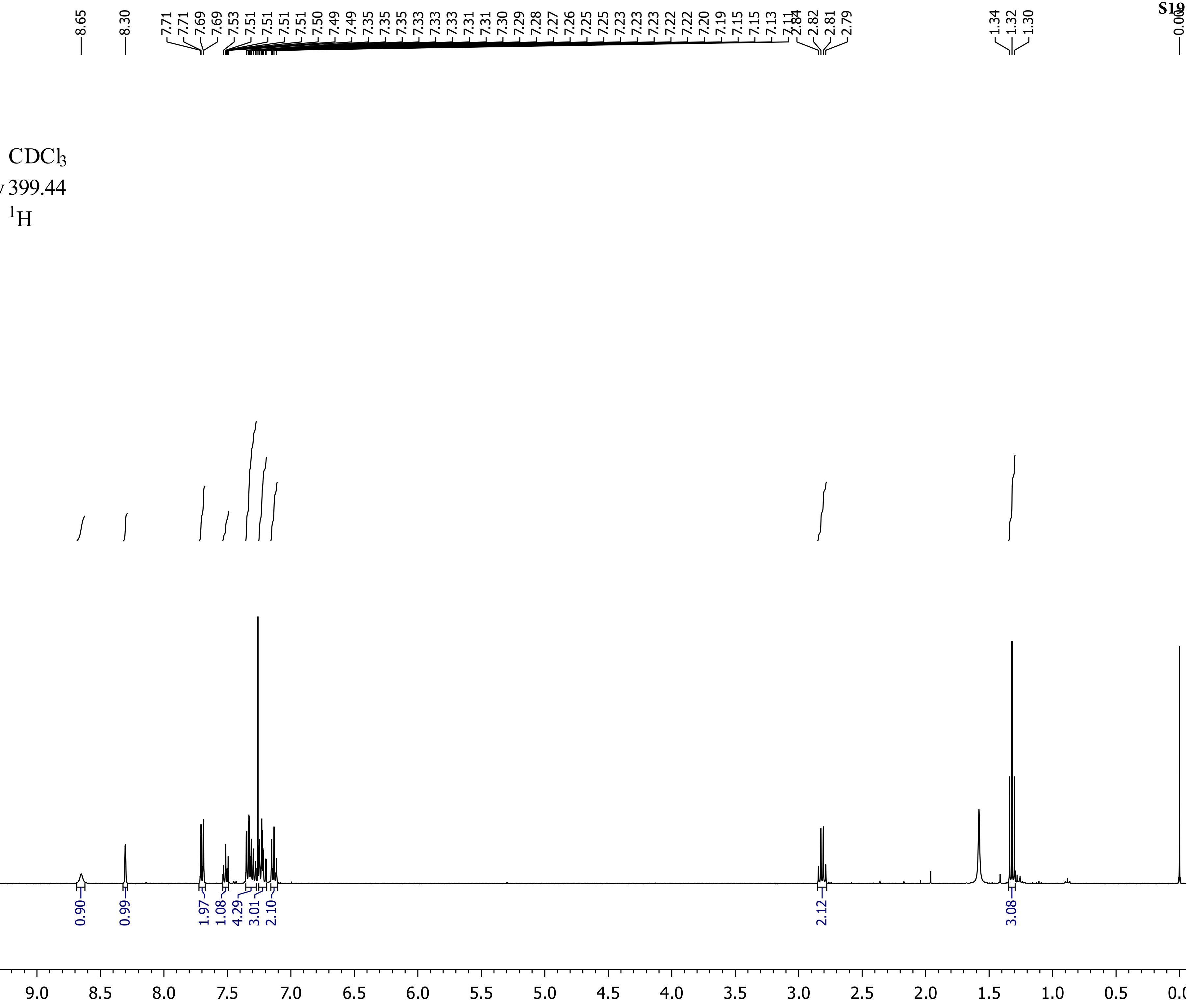

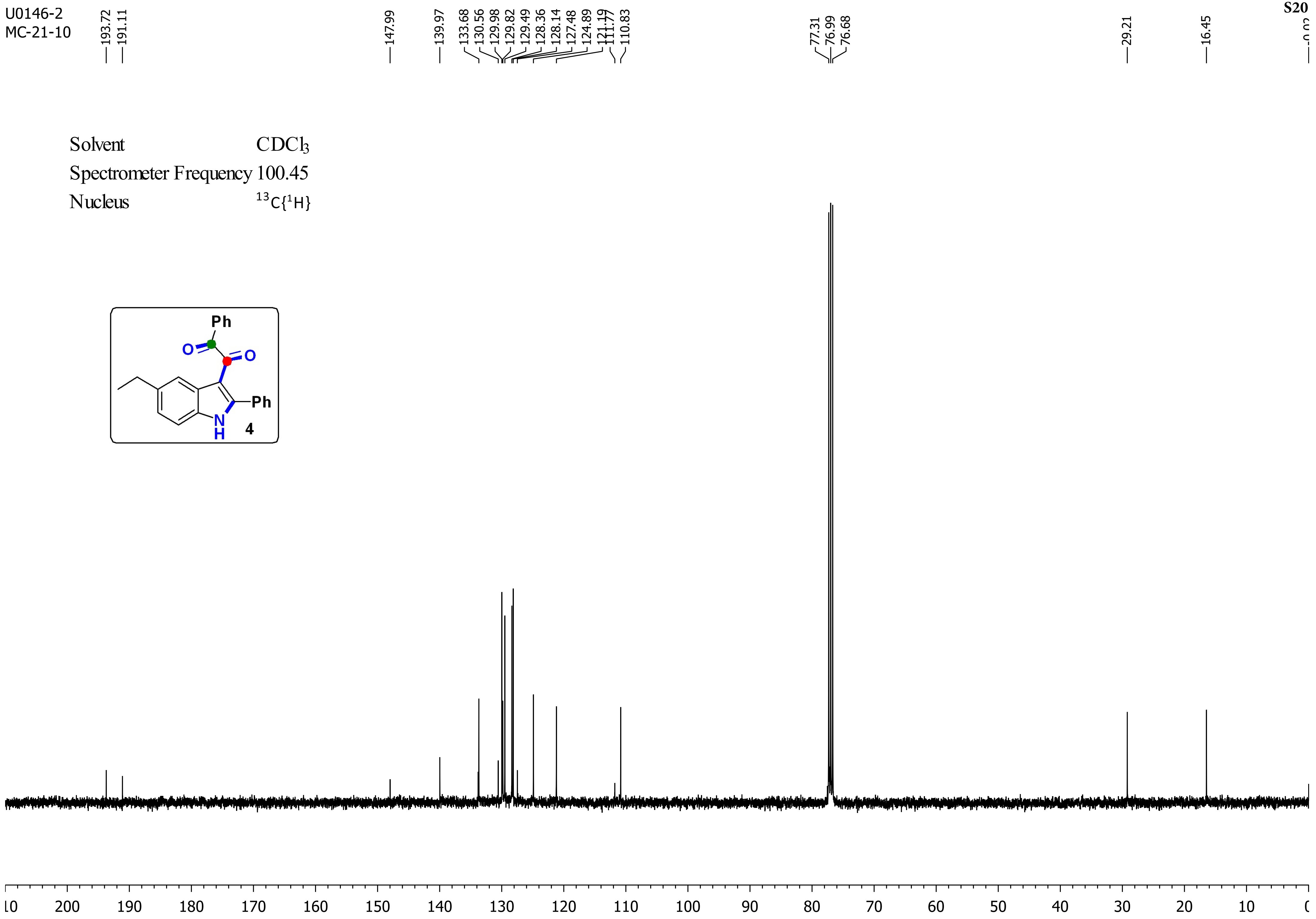
U0146-2
MC-21-10
193.72
191.11
147.99
139.97
133.68
130.56
129.98
129.82
129.49
128.36
128.14
127.48
124.89
121.19
111.77
110.83
77.31
76.99
76.68
29.21
16.45
Solvent CDCl3
Spectrometer Frequency 100.45
Nucleus 13C{1H}
Ph
O
O
Ph
N
H
4
200
190
180
170
160
150
140
130
120
110
100
90
80
70
60
50
40
30
20
10

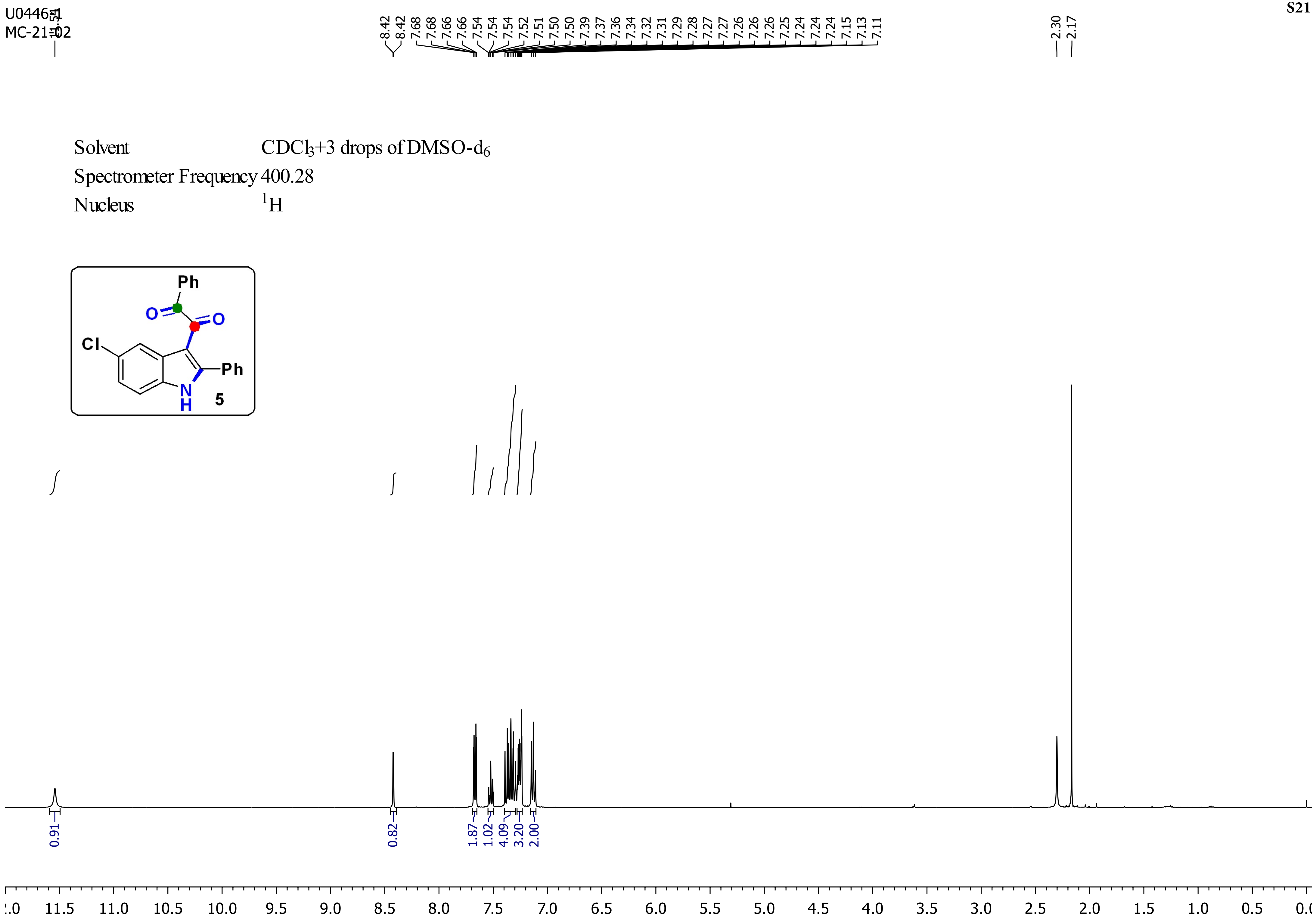
U0446[illegible]1
MC-21[illegible]02
11.54
8.42
8.42
7.68
7.68
7.66
7.66
7.54
7.54
7.54
7.52
7.51
7.50
7.50
7.39
7.37
7.36
7.34
7.32
7.31
7.29
7.28
7.27
7.27
7.26
7.26
7.26
7.25
7.24
7.24
7.24
7.15
7.13
7.11
2.30
2.17
Solvent CDCl3+3 drops of DMSO-d6
Spectrometer Frequency 400.28
Nucleus 1H
Ph
O
O
Cl
Ph
N
H
5
0.91
0.82
1.87
1.02
4.09
3.20
2.00
11.5
11.0
10.5
10.0
9.5
9.0
8.5
8.0
7.5
7.0
6.5
6.0
5.5
5.0
4.5
4.0
3.5
3.0
2.5
2.0
1.5
1.0
0.5

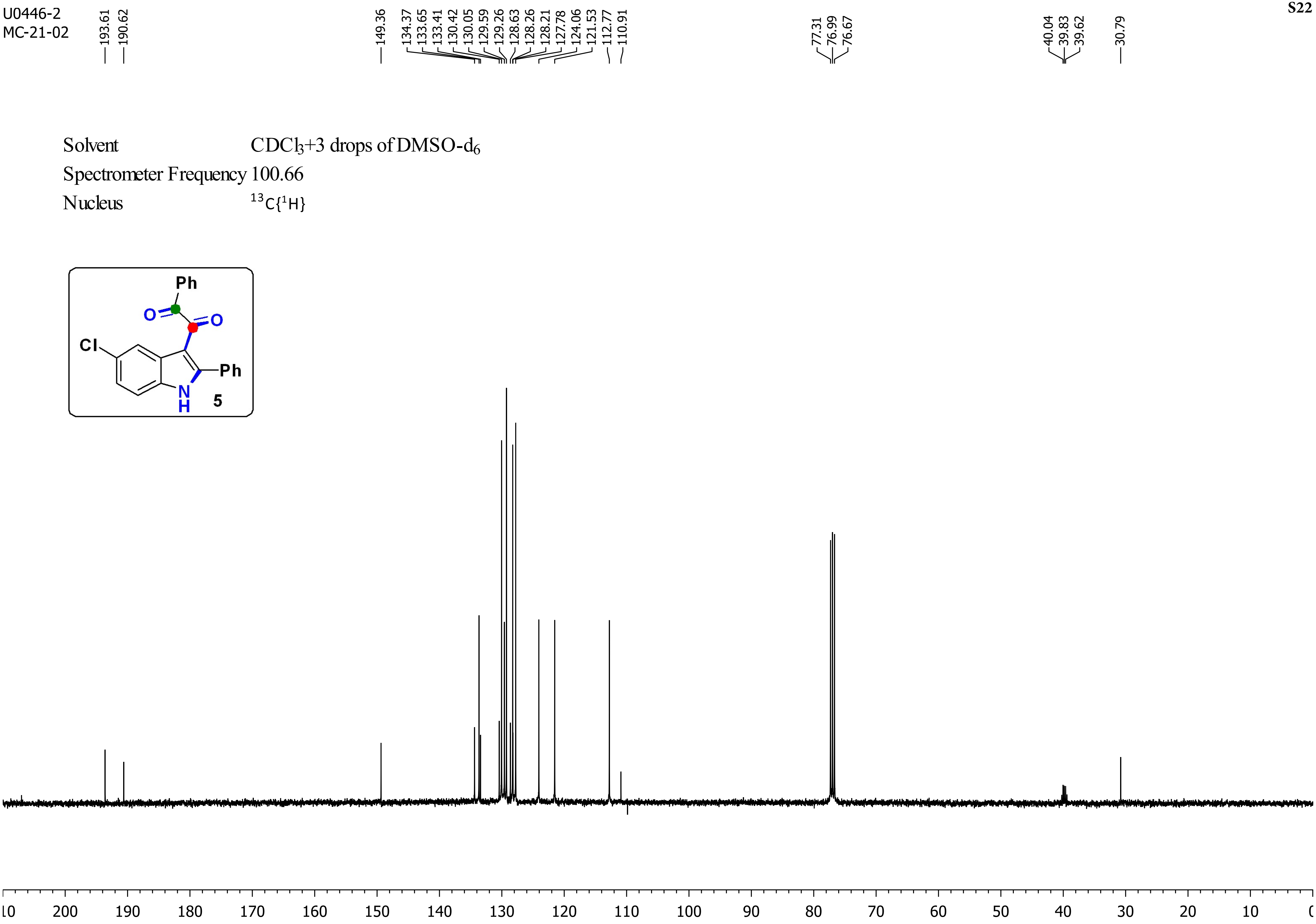
U0446-2
MC-21-02
193.61
190.62
149.36
134.37
133.65
133.41
130.42
130.05
129.59
129.26
128.63
128.26
128.21
127.78
124.06
121.53
112.77
110.91
77.31
76.99
76.67
40.04
39.83
39.62
30.79
Solvent CDCl3+3 drops of DMSO-d6
Spectrometer Frequency 100.66
Nucleus 13C{1H}
Ph
O
O
Cl
Ph
N
H
5
200
190
180
170
160
150
140
130
120
110
100
90
80
70
60
50
40
30
20
10

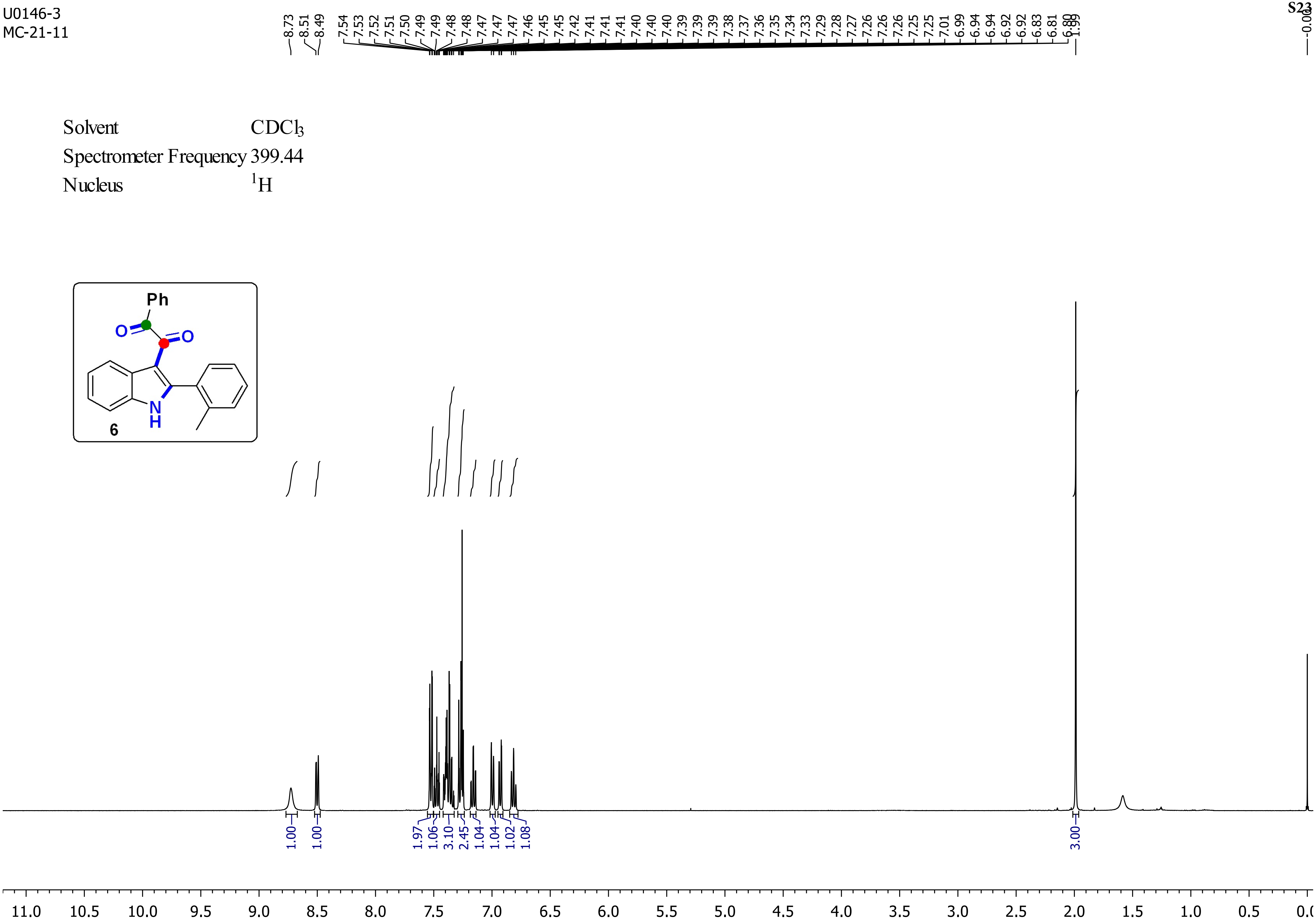
U0146-3
MC-21-11
8.73 8.51 8.49 7.54 7.53 7.52 7.51 7.50 7.49 7.49 7.48 7.48 7.47 7.47 7.47 7.46 7.45 7.45 7.42 7.41 7.41 7.41 7.40 7.40 7.40 7.39 7.39 7.39 7.38 7.37 7.36 7.35 7.34 7.33 7.29 7.28 7.27 7.26 7.26 7.26 7.25 7.25 7.01 6.99 6.94 6.94 6.92 6.92 6.83 6.81 6.80 1.99
Solvent CDCl3
Spectrometer Frequency 399.44
Nucleus 1H
Ph
O
O
N
H
6
1.00 1.00 1.97 1.06 3.10 2.45 1.04 1.04 1.02 1.08 3.00
11.0 10.5 10.0 9.5 9.0 8.5 8.0 7.5 7.0 6.5 6.0 5.5 5.0 4.5 4.0 3.5 3.0 2.5 2.0 1.5 1.0 0.5 0.0

U0146-4
MC-21-11

Solvent CDCl$_3$
Spectrometer Frequency 100.45
Nucleus $^{13}C\{^1H\}$

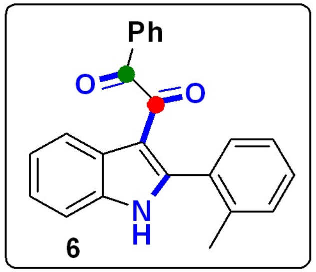

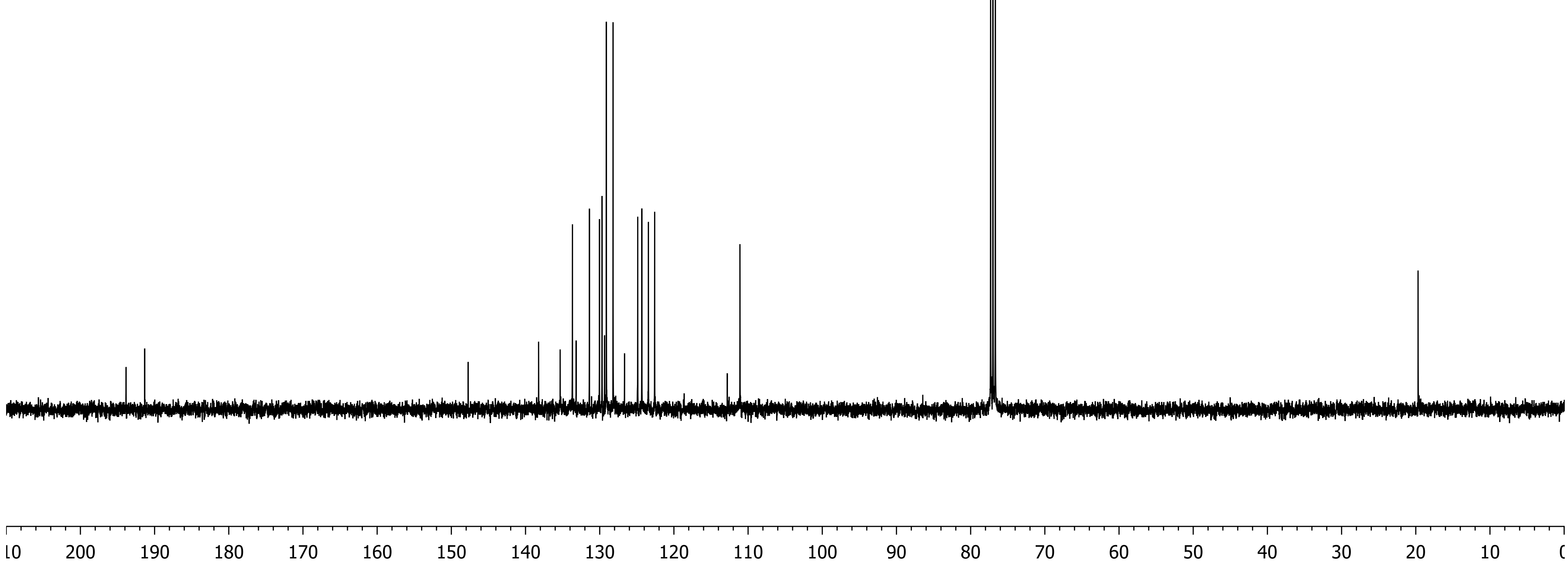

U0135-9
MC-21-9

Solvent	$CDCl_3$
Spectrometer Frequency	399.44
Nucleus	^1H

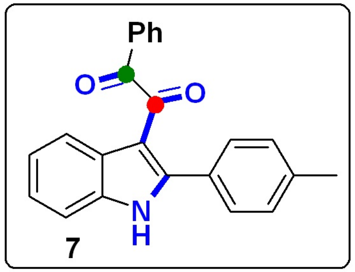

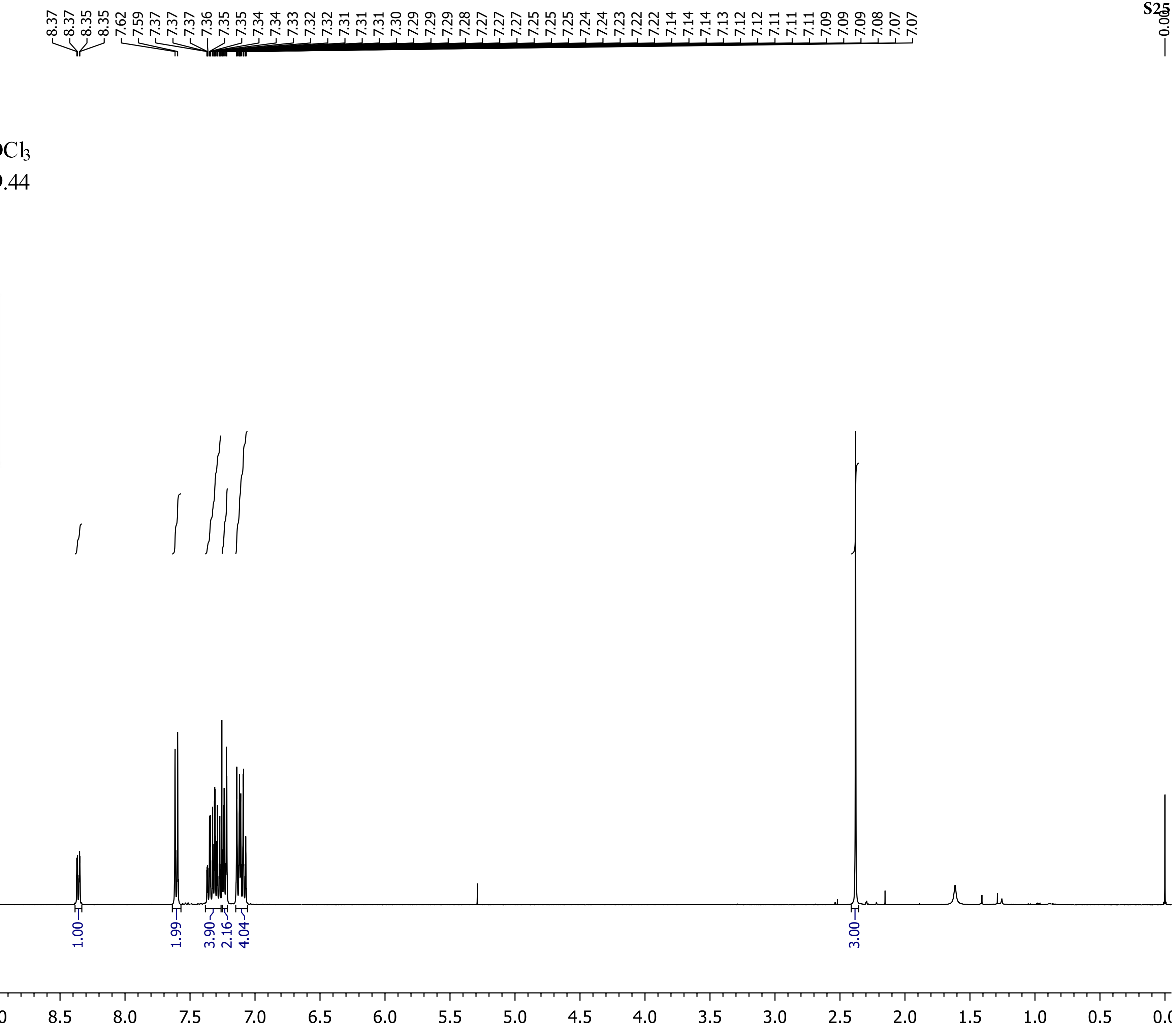

U0135-10
MC-21-9

Solvent	$CDCl_3$
Spectrometer Frequency	100.45
Nucleus	$^{13}C\{^1H\}$

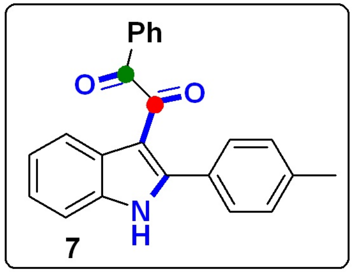

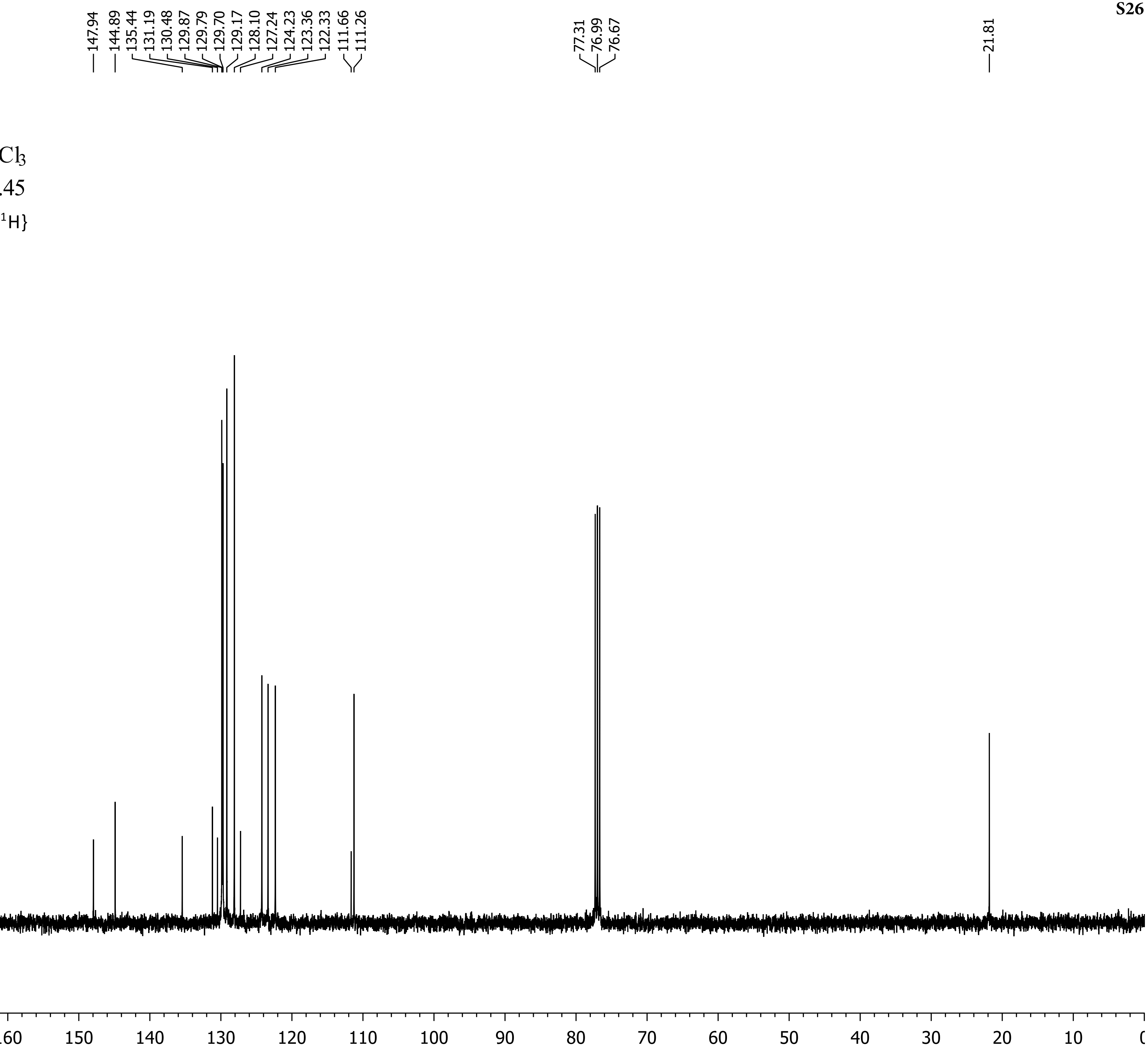

U0135-5
MC-21-7

Solvent CDCl$_3$
Spectrometer Frequency 399.44
Nucleus ^{1}H

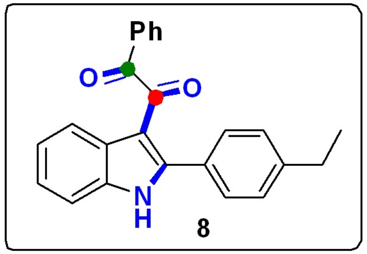

8

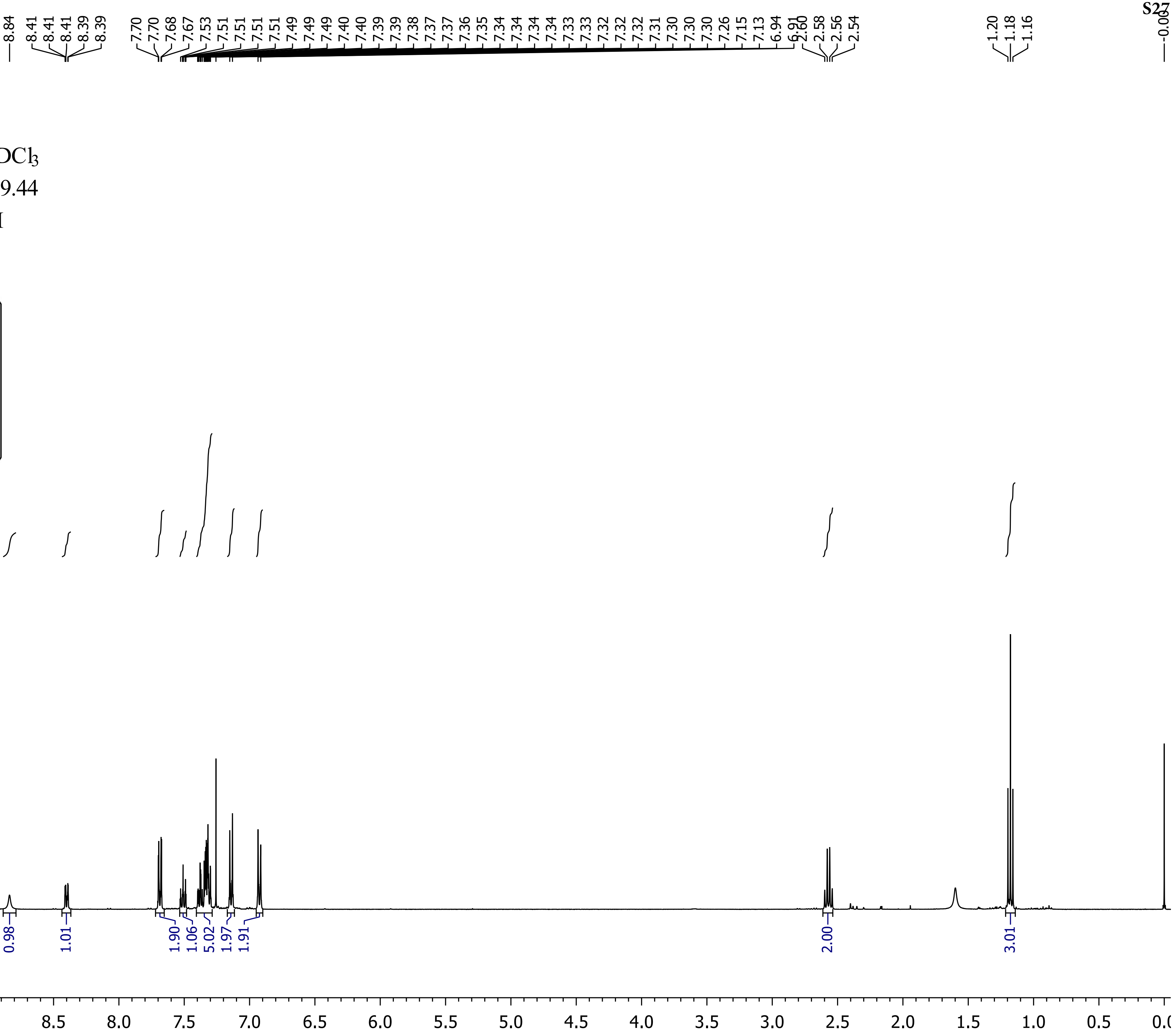

U0135-6
MC-21-7

Solvent	$CDCl_3$
Spectrometer Frequency	100.45
Nucleus	$^{13}C\{^1H\}$

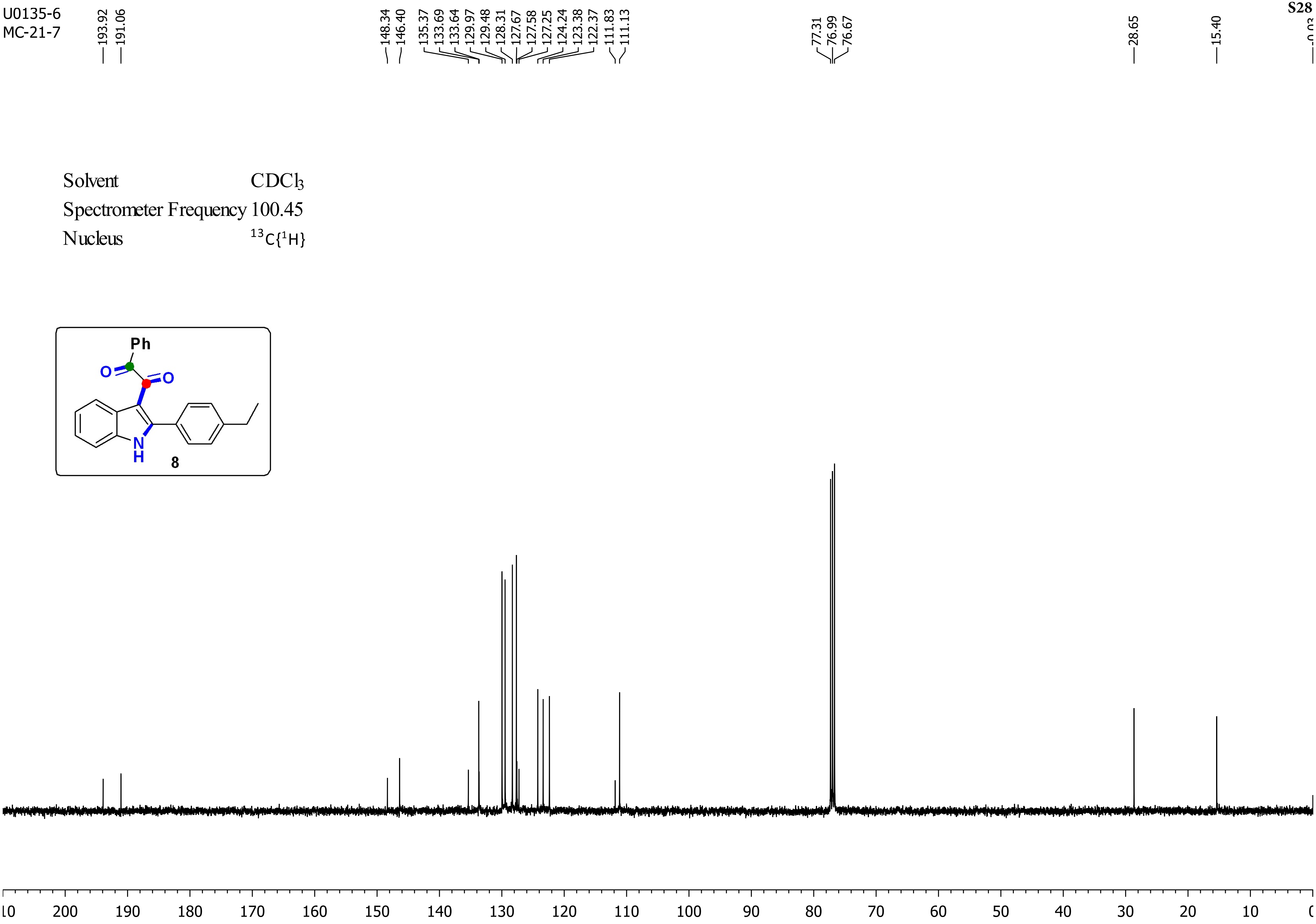

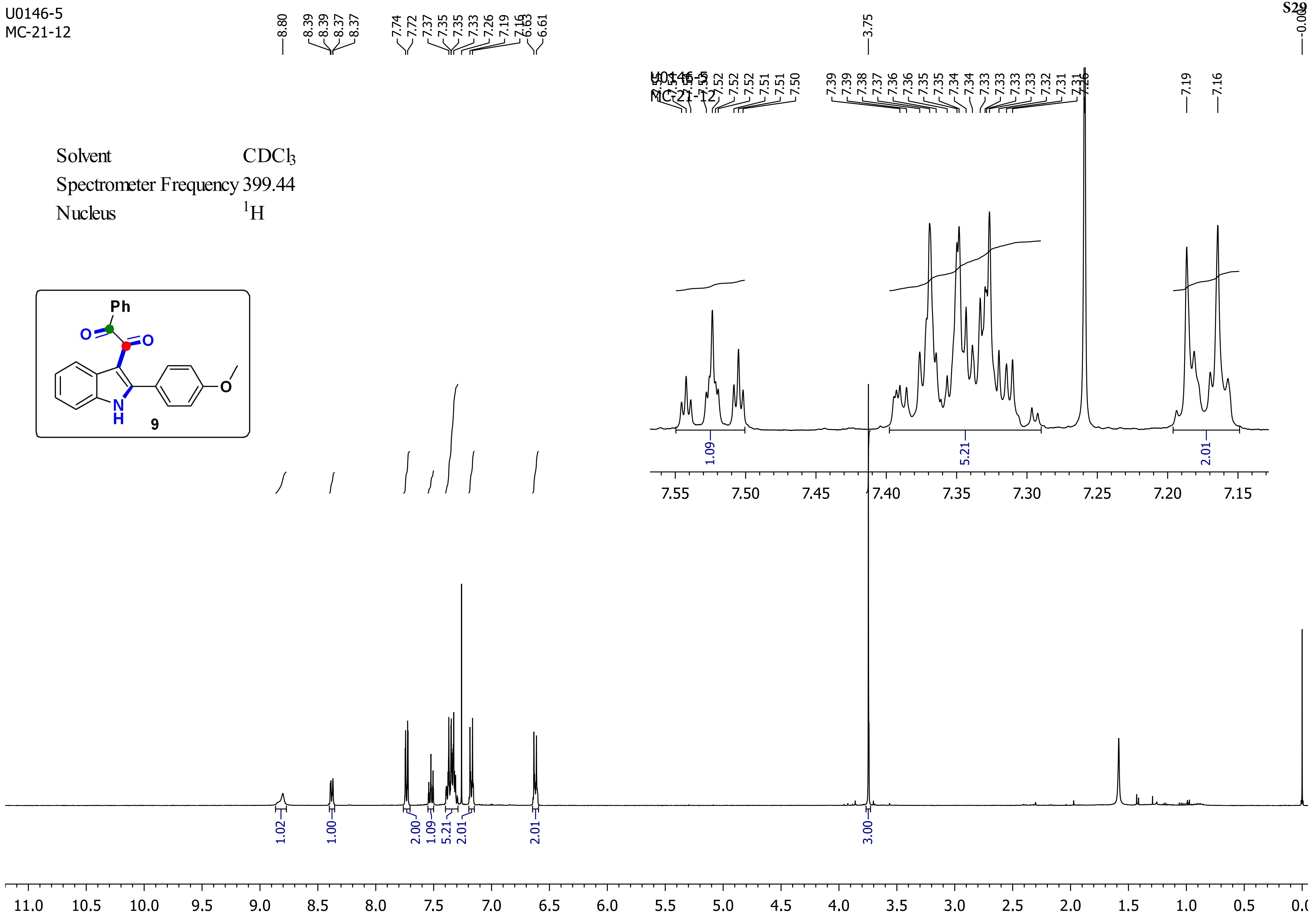
U0146-5
MC-21-12
8.80
8.39
8.39
8.37
8.37
7.74
7.72
7.37
7.35
7.35
7.33
7.26
7.19
7.16
6.63
6.61
3.75
-0.00
Solvent CDCl3
Spectrometer Frequency 399.44
Nucleus 1H
Ph
O
O
N
H
O
9
1.09
5.21
2.01
7.55
7.50
7.45
7.40
7.35
7.30
7.25
7.20
7.15
1.02
1.00
2.00
1.09
5.21
2.01
2.01
3.00
11.0
10.5
10.0
9.5
9.0
8.5
8.0
7.5
7.0
6.5
6.0
5.5
5.0
4.5
4.0
3.5
3.0
2.5
2.0
1.5
1.0
0.5

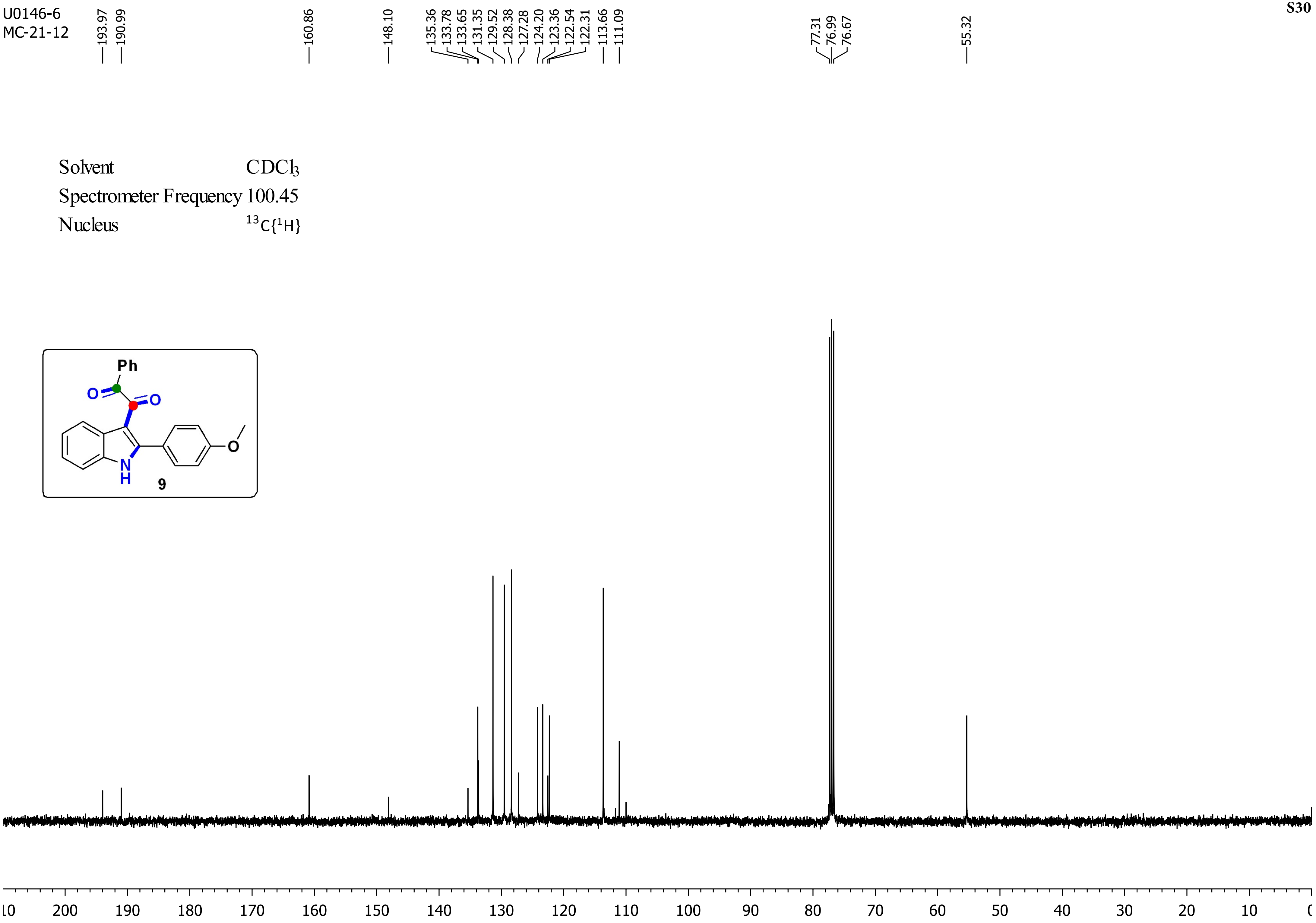
U0146-6
MC-21-12
193.97
190.99
160.86
148.10
135.36
133.78
133.65
131.35
129.52
128.38
127.28
124.20
123.36
122.54
122.31
113.66
111.09
77.31
76.99
76.67
55.32
Solvent CDCl3
Spectrometer Frequency 100.45
Nucleus 13C{1H}
Ph
O
O
N
H
O
9
200
190
180
170
160
150
140
130
120
110
100
90
80
70
60
50
40
30
20
10

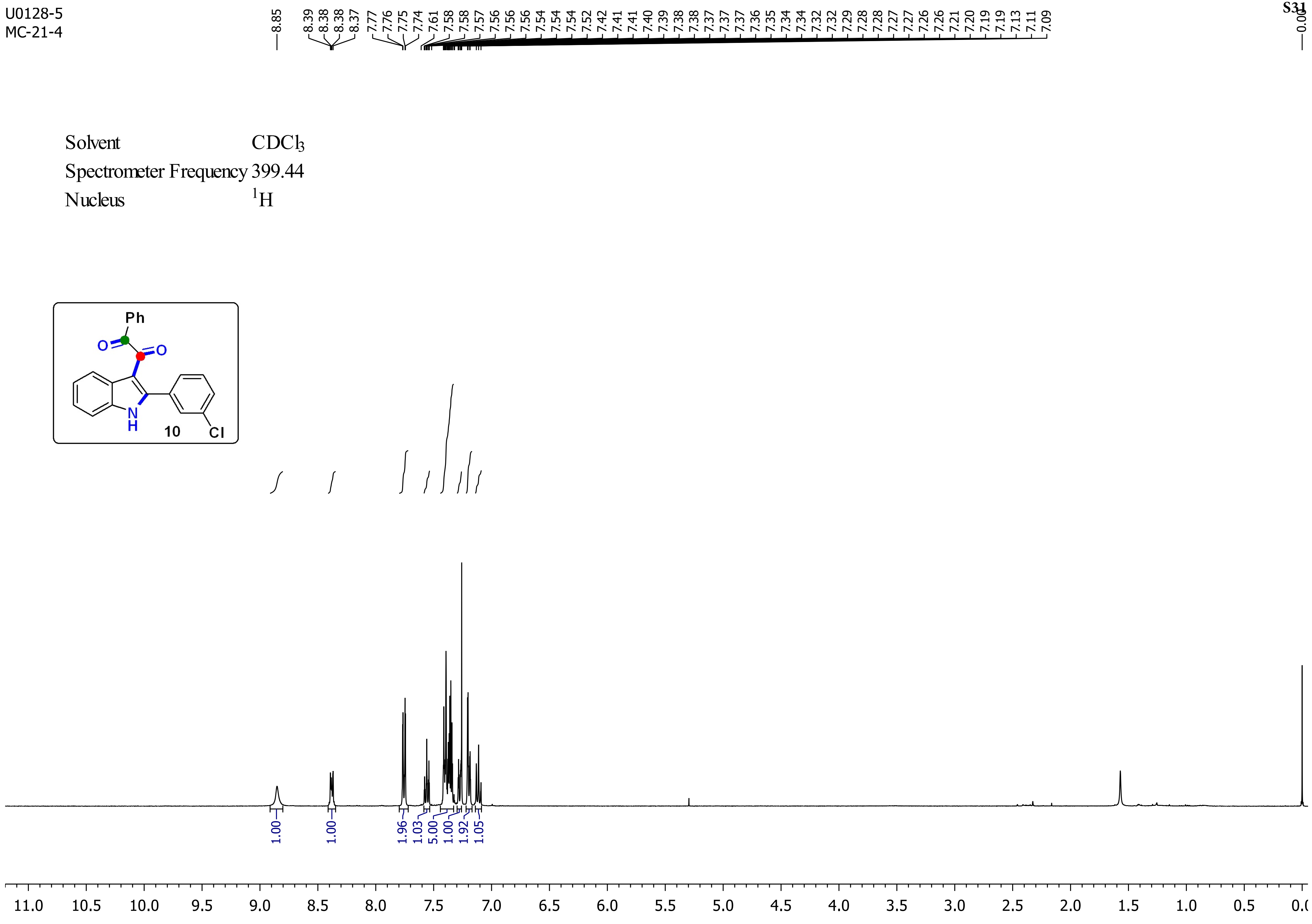
U0128-5
MC-21-4
8.85
8.39 8.38 8.38 8.37
7.77 7.76 7.75 7.74
7.61 7.58 7.58 7.57 7.56 7.56 7.56 7.54 7.54 7.54 7.52 7.42 7.41 7.41 7.40 7.39 7.38 7.38 7.37 7.37 7.37 7.36 7.35 7.34 7.34 7.32 7.32 7.29 7.28 7.28 7.27 7.27 7.26 7.26 7.21 7.20 7.19 7.19 7.13 7.11 7.09
0.00
Solvent CDCl3
Spectrometer Frequency 399.44
Nucleus 1H
Ph
O
O
N
H
10
Cl
1.00
1.00
1.96
1.03
5.00
1.00
1.92
1.05
11.0 10.5 10.0 9.5 9.0 8.5 8.0 7.5 7.0 6.5 6.0 5.5 5.0 4.5 4.0 3.5 3.0 2.5 2.0 1.5 1.0 0.5 0.0

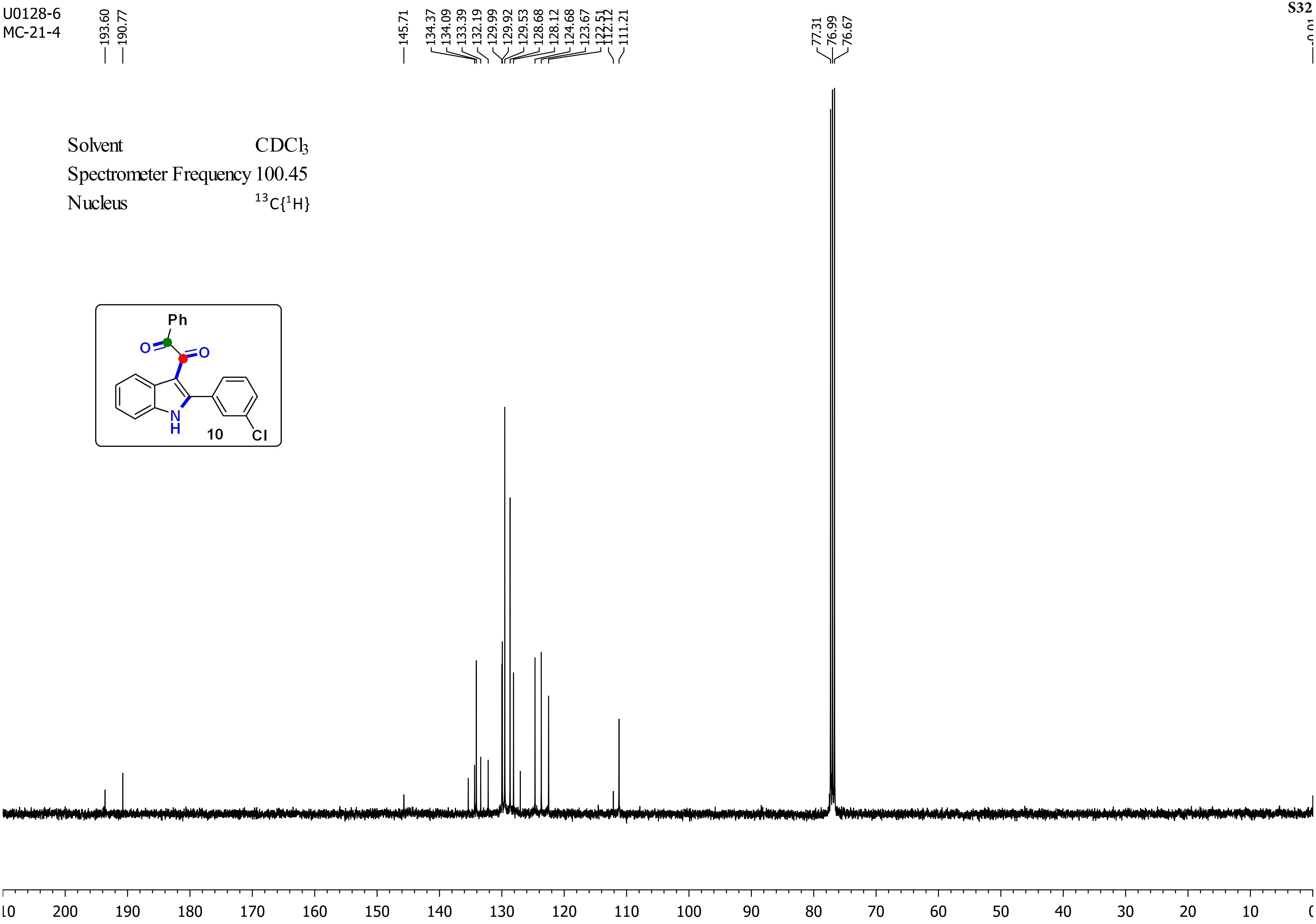
U0128-6
MC-21-4
193.60
190.77
145.71
134.37
134.09
133.39
132.19
129.99
129.92
129.53
128.68
128.12
124.68
123.67
122.51
112.12
111.21
77.31
76.99
76.67
Solvent CDCl3
Spectrometer Frequency 100.45
Nucleus 13C{1H}
200
190
180
170
160
150
140
130
120
110
100
90
80
70
60
50
40
30
20
10
Ph
O
O
N
H
10
Cl

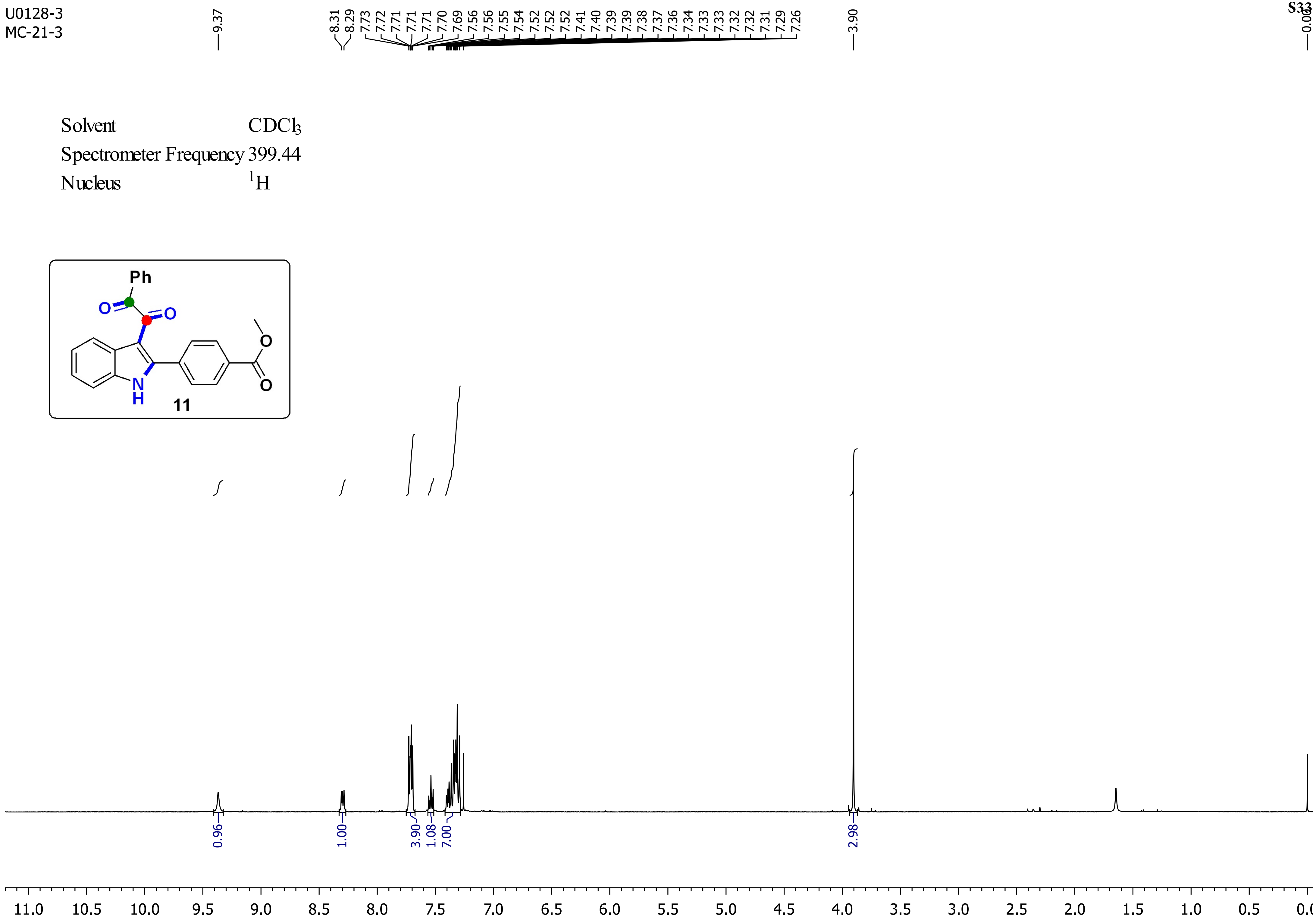
U0128-3
MC-21-3
Solvent CDCl3
Spectrometer Frequency 399.44
Nucleus 1H
Ph
O
O
O
O
N
H
11
9.37
8.31 8.29 7.73 7.72 7.71 7.71 7.71 7.70 7.69 7.56 7.56 7.55 7.54 7.52 7.52 7.52 7.41 7.40 7.39 7.39 7.38 7.37 7.36 7.34 7.33 7.33 7.32 7.32 7.31 7.29 7.26
3.90
0.00
0.96
1.00
3.90
1.08
7.00
2.98
11.0 10.5 10.0 9.5 9.0 8.5 8.0 7.5 7.0 6.5 6.0 5.5 5.0 4.5 4.0 3.5 3.0 2.5 2.0 1.5 1.0 0.5 0.0

U0128-4
MC-21-3

Solvent	$CDCl_3$
Spectrometer Frequency	100.45
Nucleus	$^{13}C\{^1H\}$

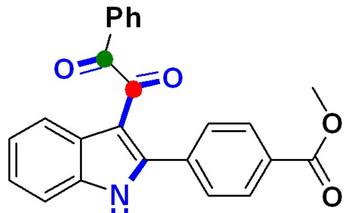

11

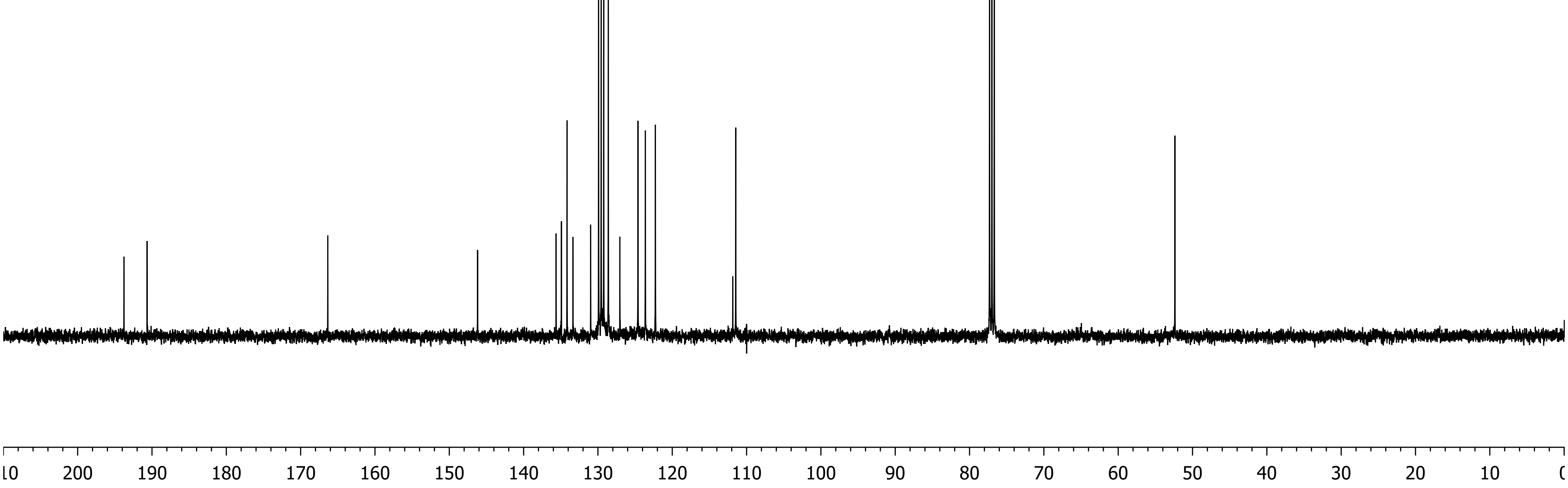

U0208-1
MC-21-15

Solvent CDCl$_3$
Spectrometer Frequency 399.44
Nucleus ^{1}H

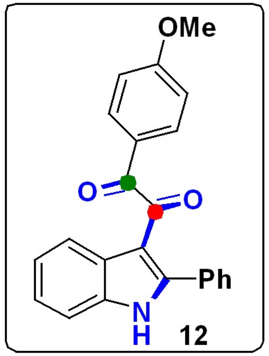

12

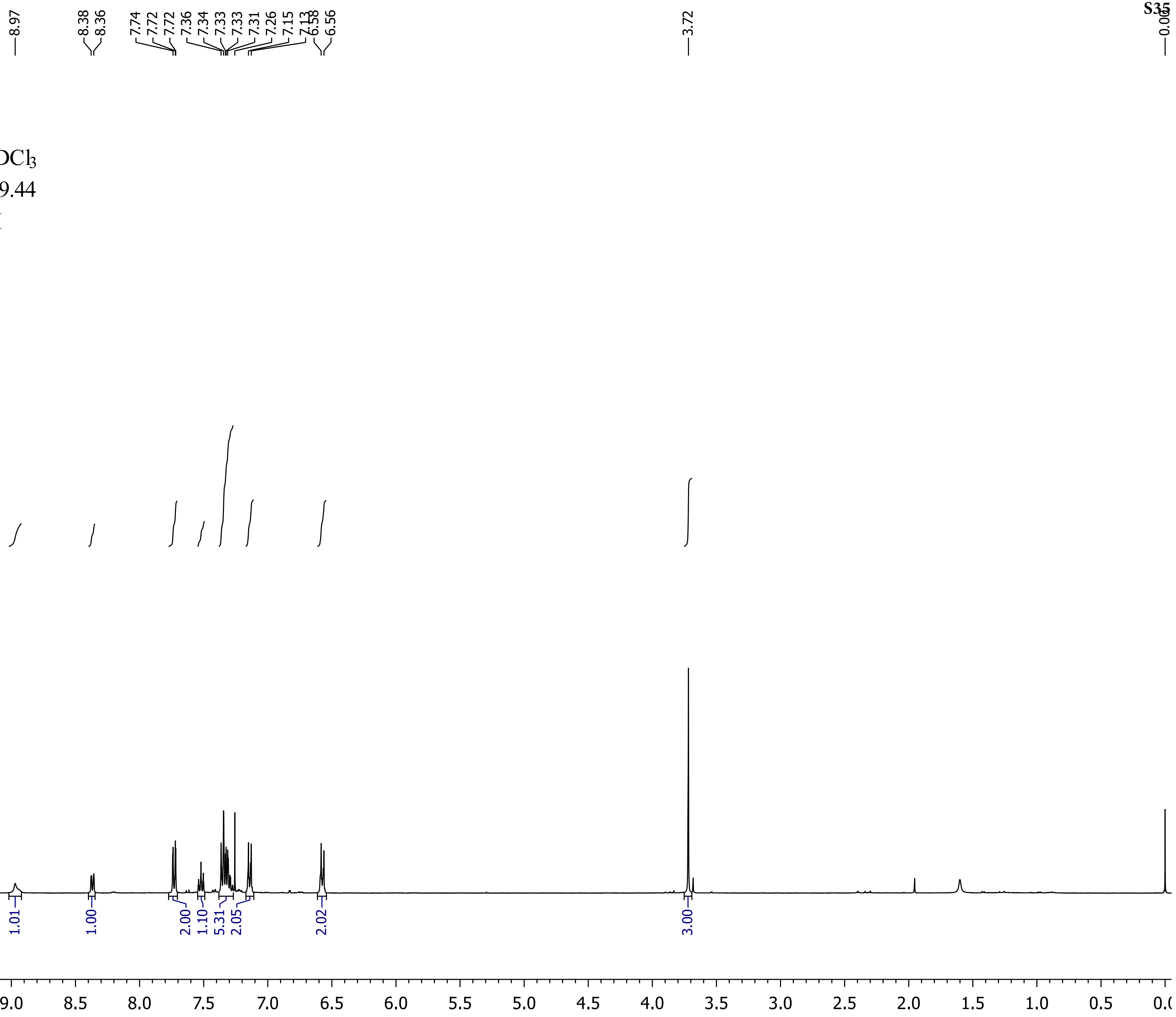

U0208-2
MC-21-15

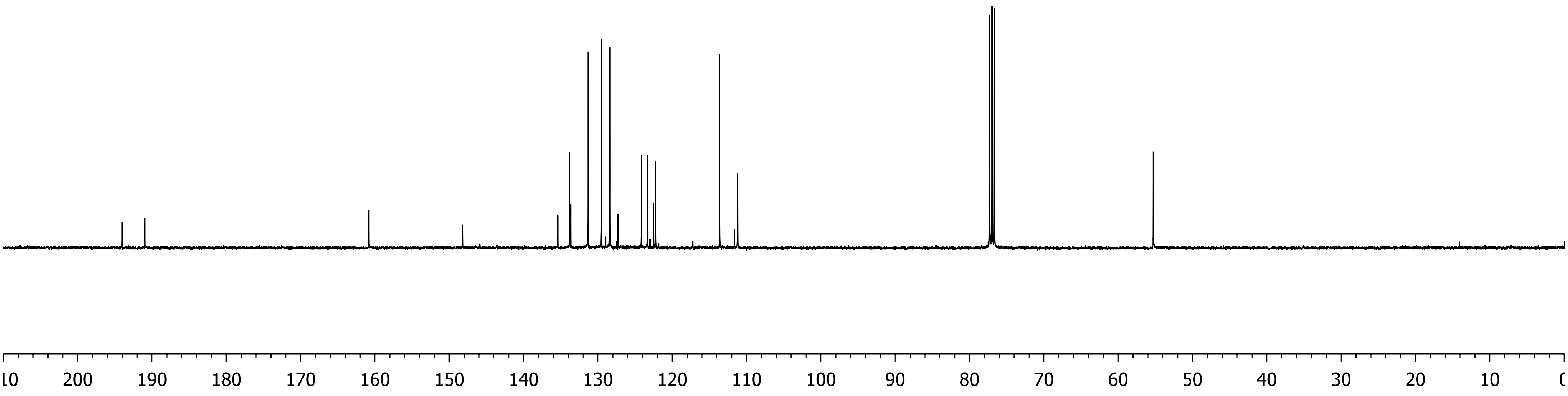

Solvent	$CDCl_3$
Spectrometer Frequency	100.45
Nucleus	$^{13}C\{^1H\}$

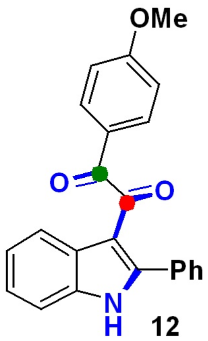

12

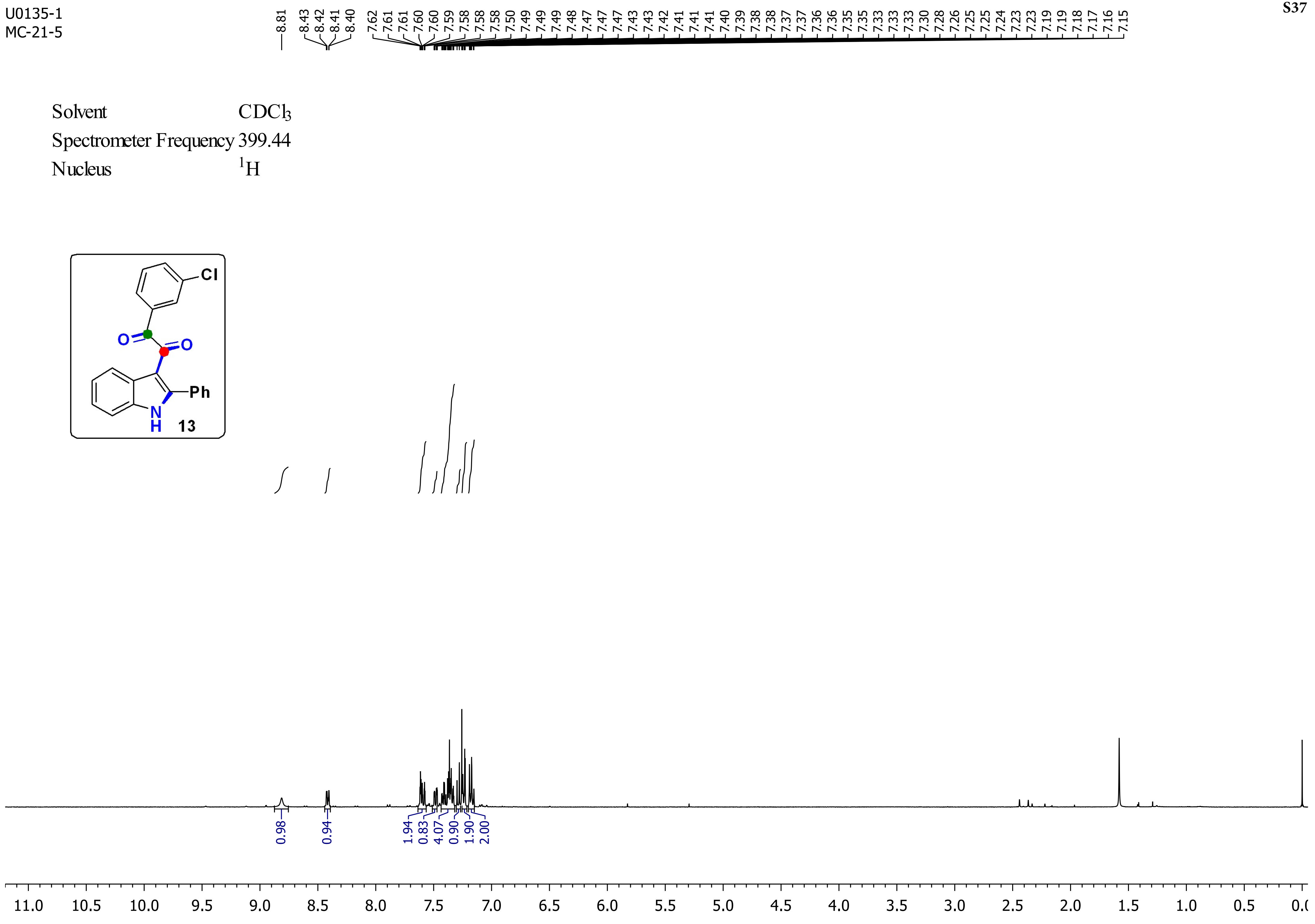
U0135-1
MC-21-5
8.81
8.43 8.42 8.41 8.40
7.62 7.61 7.61 7.60 7.60 7.59 7.58 7.58 7.58 7.50 7.49 7.49 7.49 7.48 7.47 7.47 7.47 7.43 7.43 7.42 7.41 7.41 7.41 7.40 7.39 7.38 7.38 7.37 7.37 7.36 7.36 7.35 7.35 7.33 7.33 7.33 7.30 7.28 7.26 7.25 7.25 7.24 7.23 7.23 7.19 7.19 7.18 7.17 7.16 7.15
Solvent CDCl3
Spectrometer Frequency 399.44
Nucleus 1H
Cl
O
O
Ph
N
H
13
0.98
0.94
1.94
0.83
4.07
0.90
1.90
2.00
11.0 10.5 10.0 9.5 9.0 8.5 8.0 7.5 7.0 6.5 6.0 5.5 5.0 4.5 4.0 3.5 3.0 2.5 2.0 1.5 1.0 0.5 0.0

U0135-2
MC-21-5

Solvent	$CDCl_3$
Spectrometer Frequency	100.45
Nucleus	$^{13}C\{^1H\}$

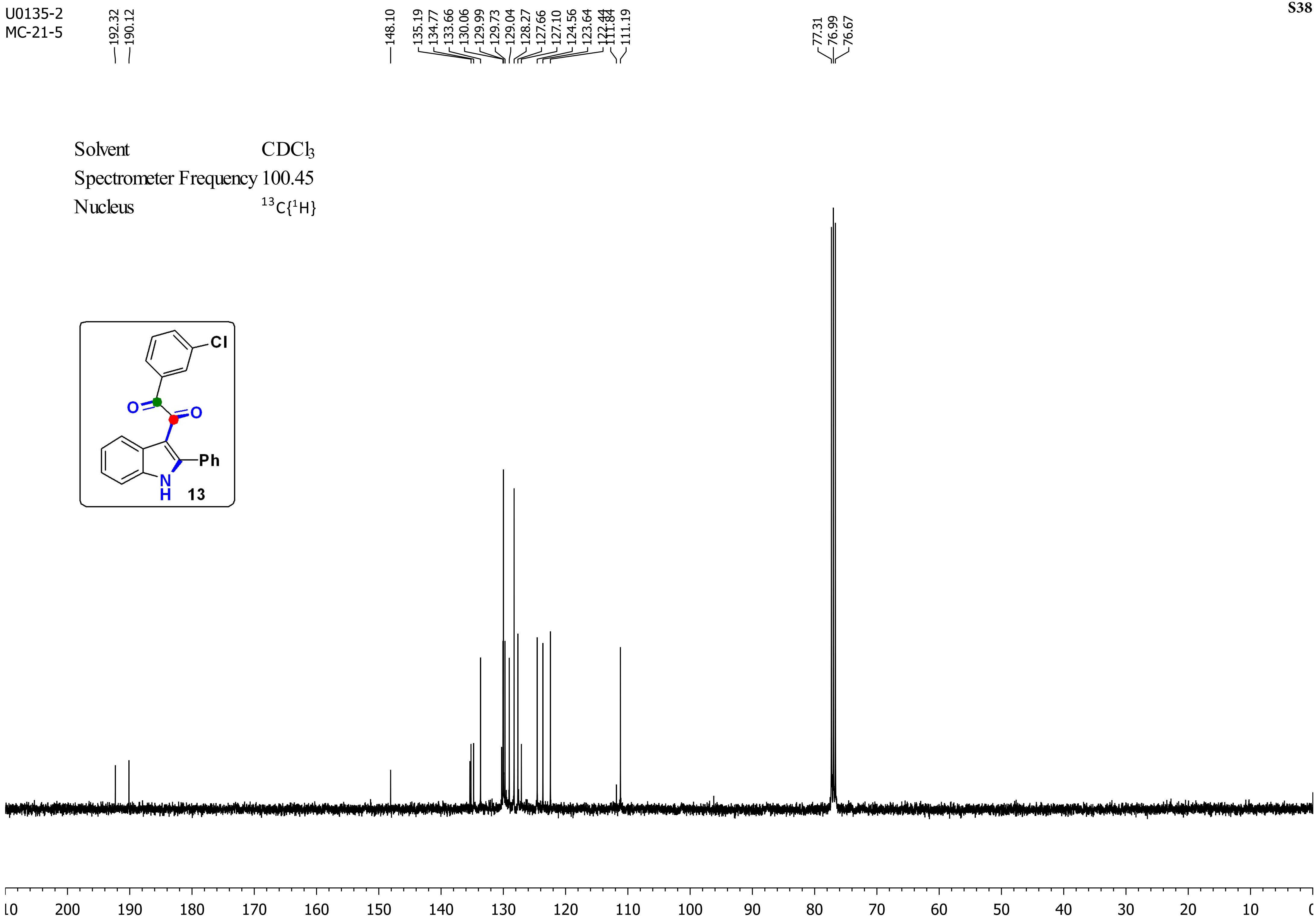

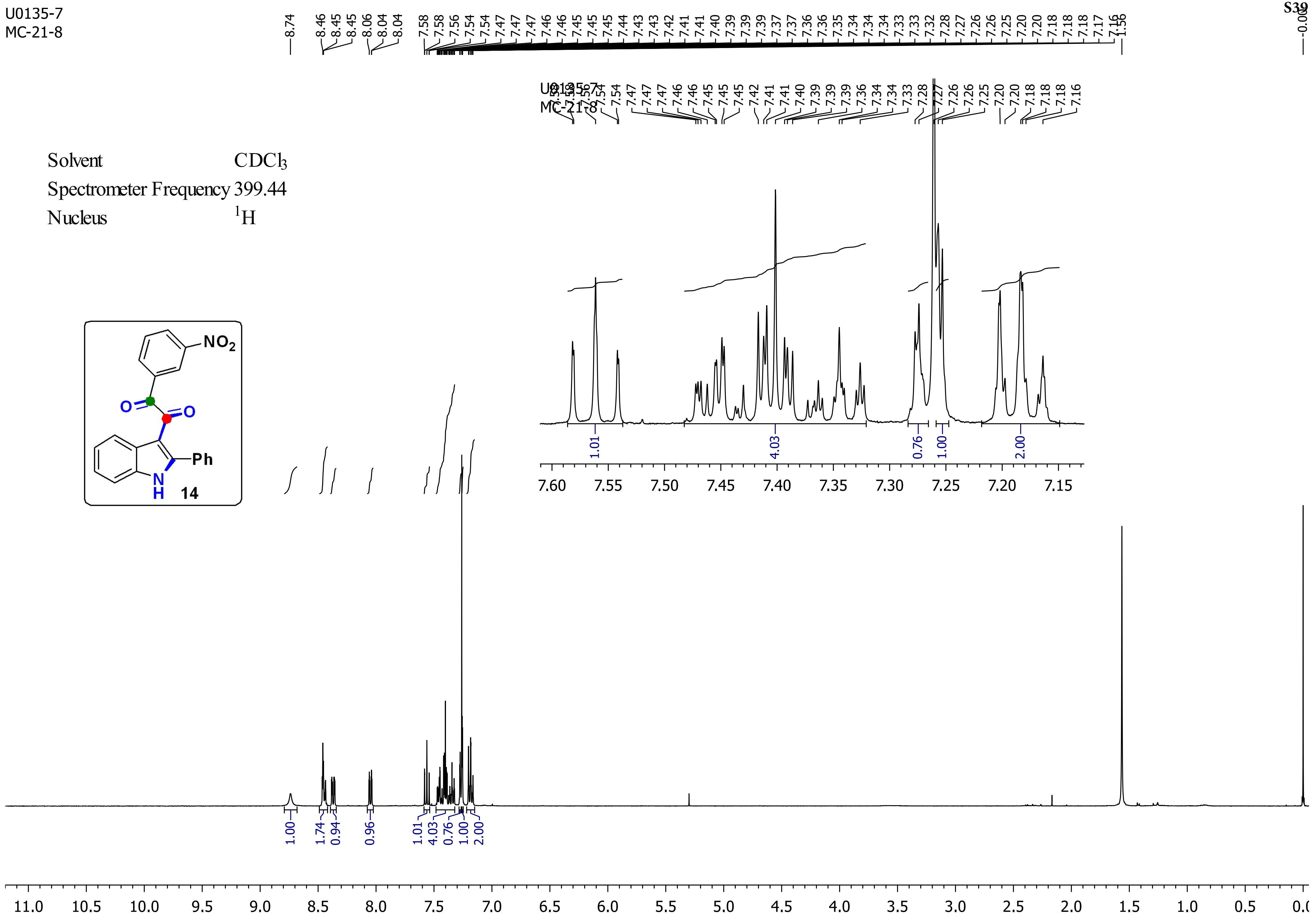
U0135-7
MC-21-8
Solvent CDCl3
Spectrometer Frequency 399.44
Nucleus 1H
NO2
O
O
Ph
N
H
14
8.74
8.46 8.45 8.45
8.06 8.04 8.04
7.58 7.58 7.56 7.54 7.54 7.47 7.47 7.47 7.46 7.46 7.45 7.45 7.45 7.44 7.43 7.43 7.42 7.41 7.41 7.40 7.39 7.39 7.39 7.37 7.37 7.36 7.36 7.35 7.34 7.34 7.34 7.33 7.33 7.32 7.28 7.27 7.26 7.26 7.25 7.20 7.20 7.18 7.18 7.18 7.17 7.16
1.56
-0.00
1.00 1.74 0.94 0.96 1.01 4.03 0.76 1.00 2.00
11.0 10.5 10.0 9.5 9.0 8.5 8.0 7.5 7.0 6.5 6.0 5.5 5.0 4.5 4.0 3.5 3.0 2.5 2.0 1.5 1.0 0.5 0.0
7.60 7.55 7.50 7.45 7.40 7.35 7.30 7.25 7.20 7.15

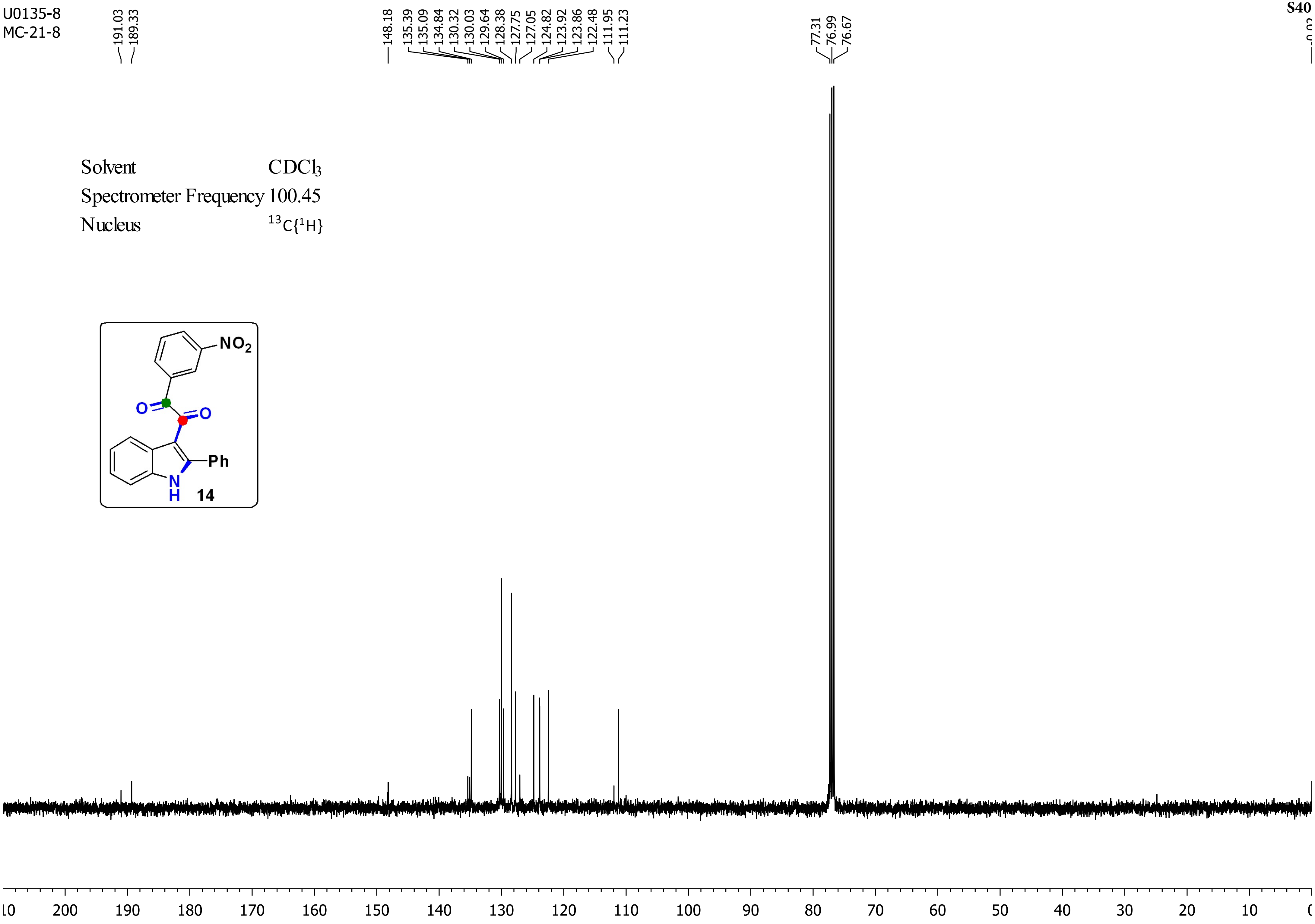
U0135-8
MC-21-8
191.03
189.33
148.18
135.39
135.09
134.84
130.32
130.03
129.64
128.38
127.75
127.05
124.82
123.92
123.86
122.48
111.95
111.23
77.31
76.99
76.67
Solvent CDCl3
Spectrometer Frequency 100.45
Nucleus 13C{1H}
NO2
O
O
Ph
N
H
14
200
190
180
170
160
150
140
130
120
110
100
90
80
70
60
50
40
30
20
10

U0146-7
MC-21-13

Solvent CDCl$_3$
Spectrometer Frequency 399.44
Nucleus ^{1}H

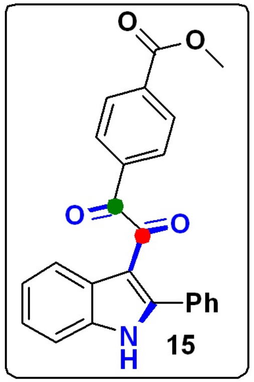

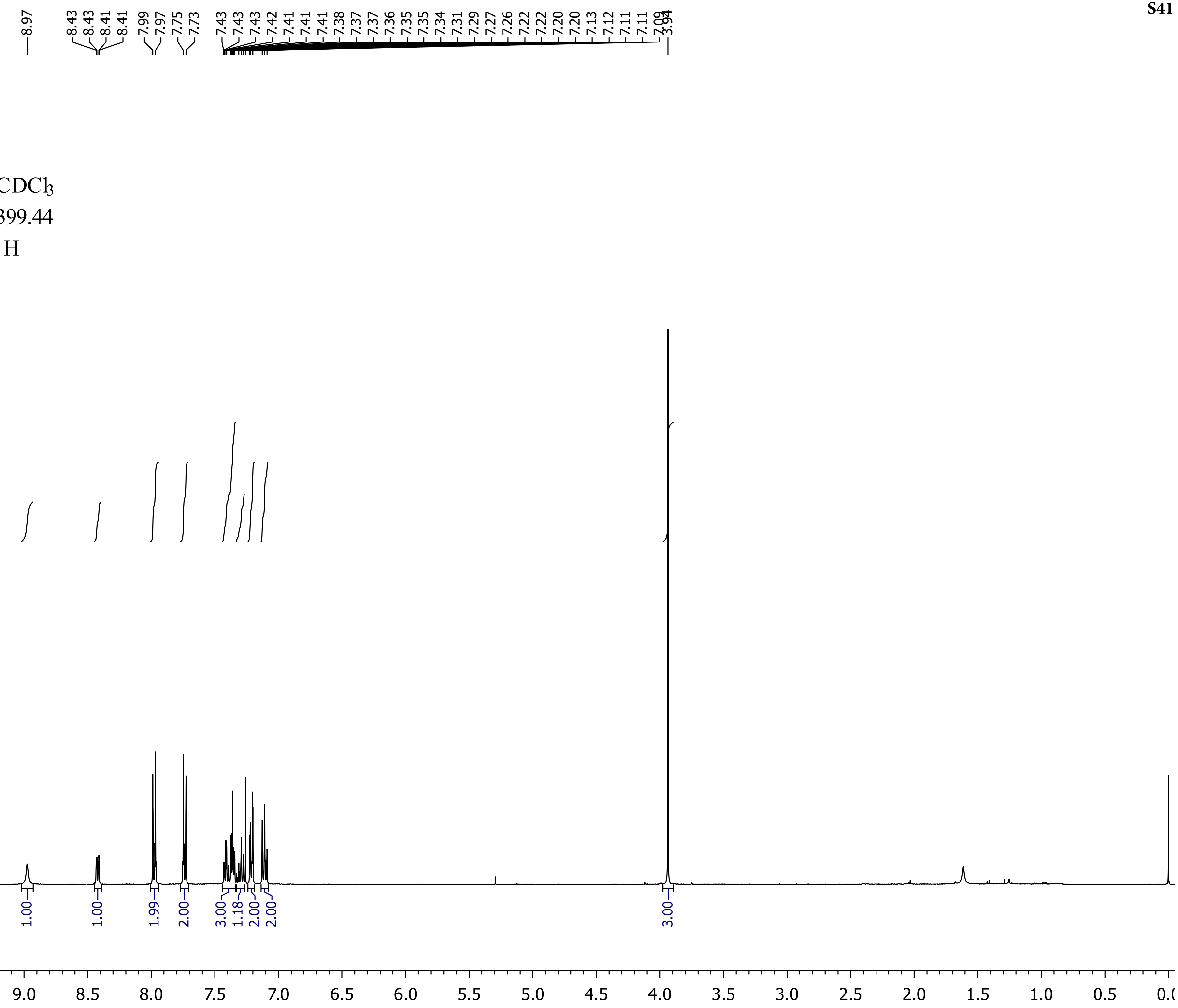

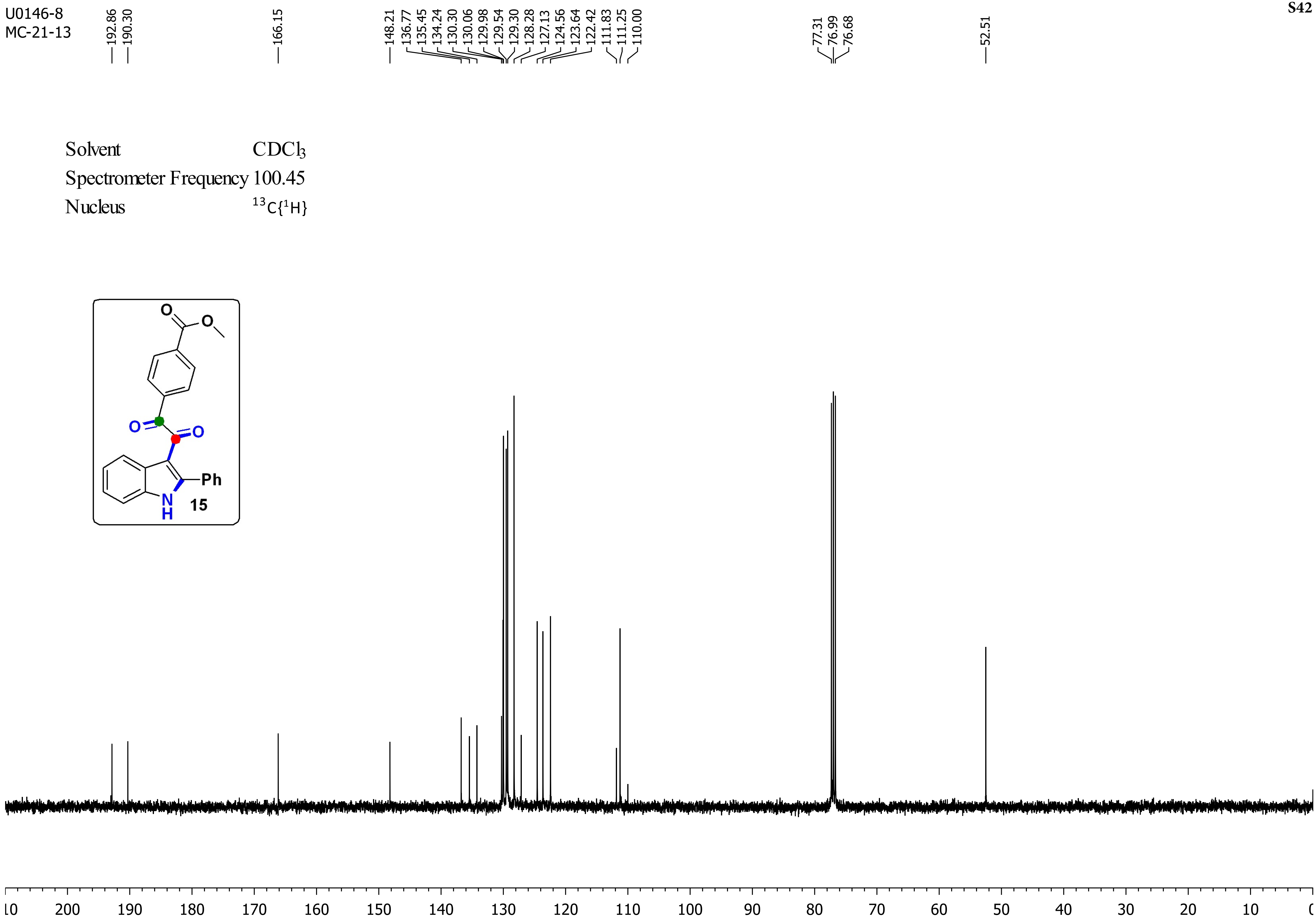

U0146-8
MC-21-13
192.86
190.30
166.15
148.21
136.77
135.45
134.24
130.30
130.06
129.98
129.54
129.30
128.28
127.13
124.56
123.64
122.42
111.83
111.25
110.00
77.31
76.99
76.68
52.51
Solvent CDCl3
Spectrometer Frequency 100.45
Nucleus 13C{1H}
O
O
O
O
Ph
N
H
15
200
190
180
170
160
150
140
130
120
110
100
90
80
70
60
50
40
30
20
10

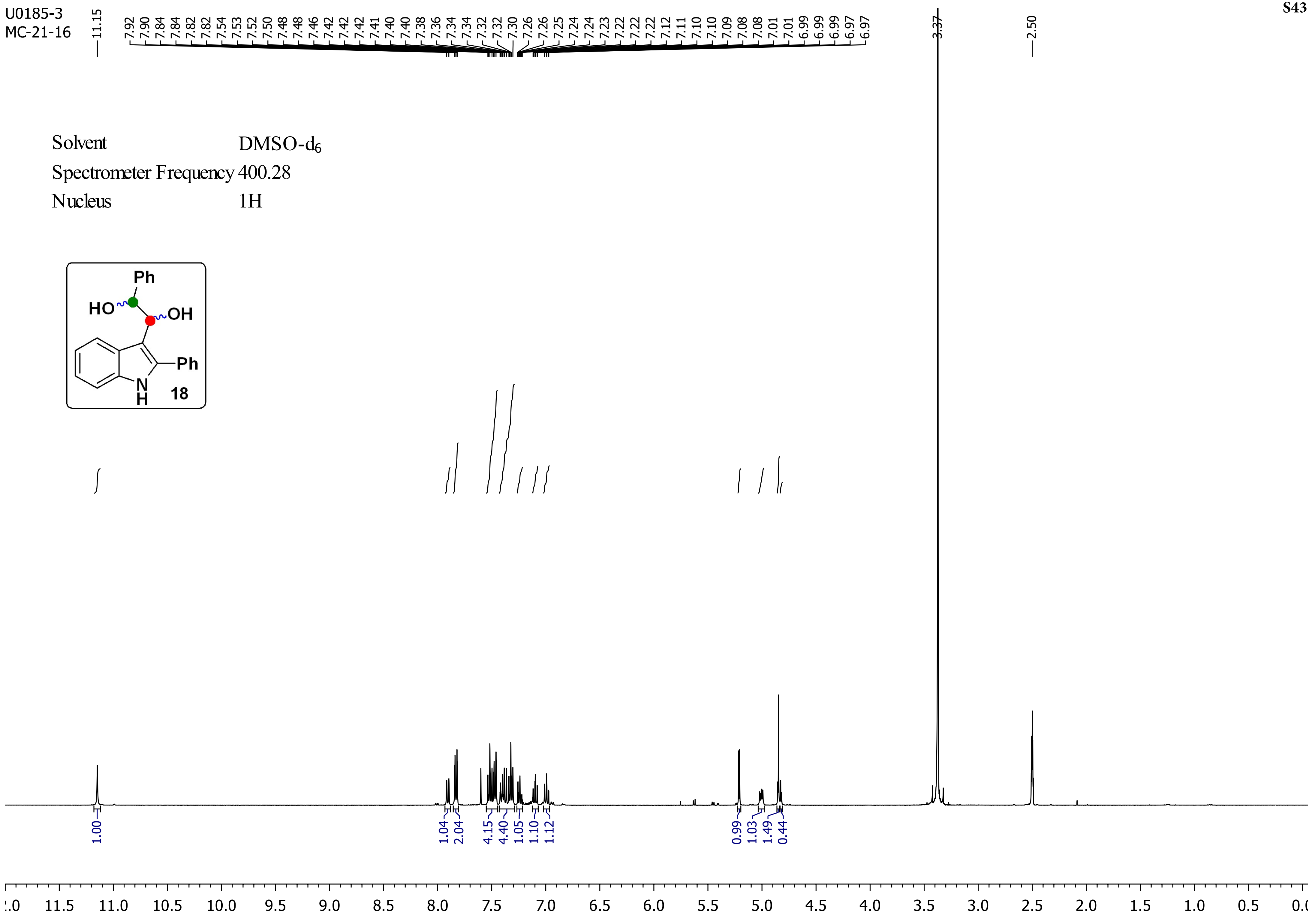
U0185-3
MC-21-16
11.15
7.92 7.90 7.84 7.84 7.82 7.82 7.54 7.53 7.52 7.50 7.48 7.48 7.46 7.42 7.42 7.42 7.41 7.40 7.40 7.38 7.36 7.34 7.34 7.32 7.32 7.30 7.26 7.26 7.25 7.24 7.24 7.23 7.22 7.22 7.22 7.12 7.11 7.10 7.10 7.09 7.08 7.08 7.01 7.01 6.99 6.99 6.97 6.97
3.37
2.50
Solvent DMSO-d6
Spectrometer Frequency 400.28
Nucleus 1H
Ph
HO
OH
Ph
N
H
18
1.00
1.04
2.04
4.15
4.40
1.05
1.10
1.12
0.99
1.03
1.49
0.44
11.5 11.0 10.5 10.0 9.5 9.0 8.5 8.0 7.5 7.0 6.5 6.0 5.5 5.0 4.5 4.0 3.5 3.0 2.5 2.0 1.5 1.0 0.5

U0185-4
MC-21-16

Solvent DMSO-d_6
Spectrometer Frequency 100.66
Nucleus $^{13}C\{^1H\}$

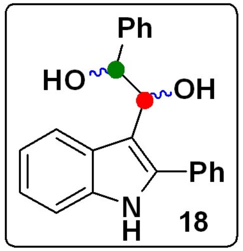

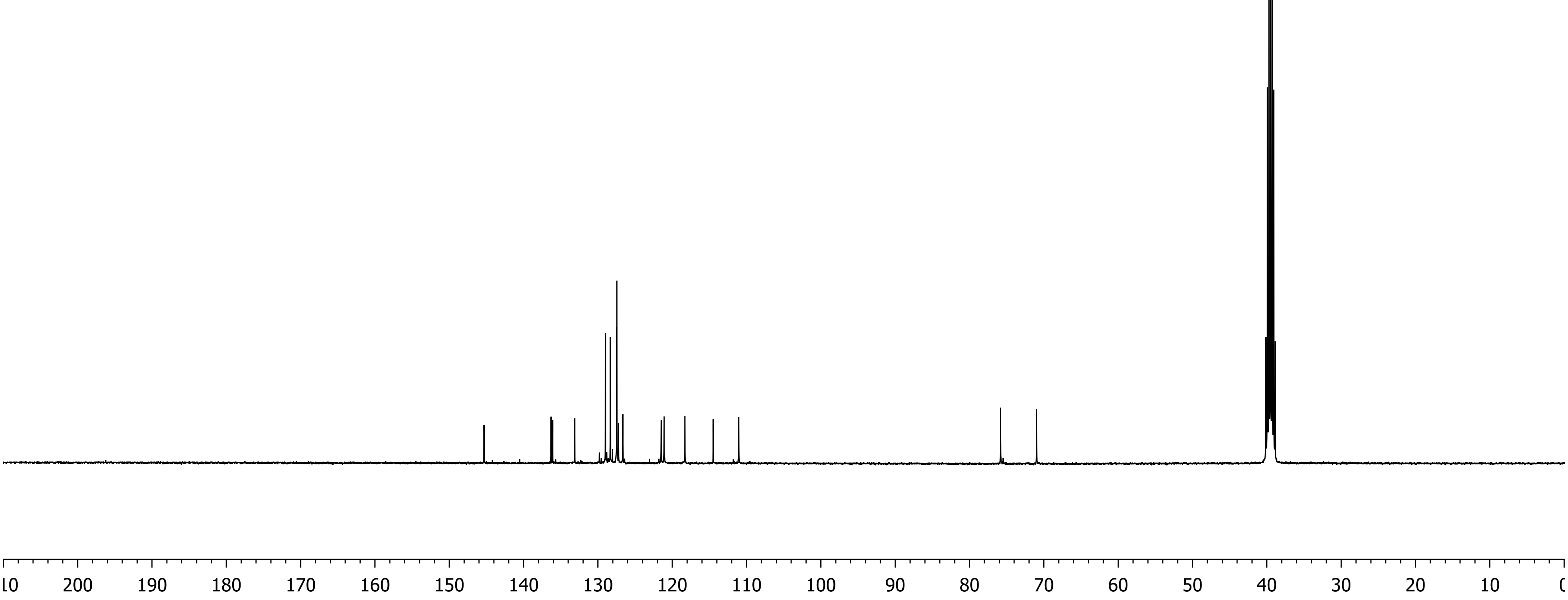

Supplement: Supplementary file 1 [file jo5c01007_si_001.pdf]
